# Review on Blueprint of Designing Anti-Wetting Polymeric Membrane Surfaces for Enhanced Membrane Distillation Performance

**DOI:** 10.3390/polym12010023

**Published:** 2019-12-20

**Authors:** Saikat Sinha Ray, Hyung-Kae Lee, Young-Nam Kwon

**Affiliations:** School of Urban and Environmental Engineering, Ulsan National Institute of Science and Technology (UNIST), Ulsan 44919, Korea; ssinharay6@gmail.com (S.S.R.); sosky@unist.ac.kr (H.-K.L.)

**Keywords:** membrane distillation, anti-wetting, superhydrophobic, fouling, desalination

## Abstract

Recently, membrane distillation (MD) has emerged as a versatile technology for treating saline water and industrial wastewater. However, the long-term use of MD wets the polymeric membrane and prevents the membrane from working as a semi-permeable barrier. Currently, the concept of antiwetting interfaces has been utilized for reducing the wetting issue of MD. This review paper discusses the fundamentals and roles of surface energy and hierarchical structures on both the hydrophobic characteristics and wetting tolerance of MD membranes. Designing stable antiwetting interfaces with their basic working principle is illustrated with high scientific discussions. The capability of antiwetting surfaces in terms of their self-cleaning properties has also been demonstrated. This comprehensive review paper can be utilized as the fundamental basis for developing antiwetting surfaces to minimize fouling, as well as the wetting issue in the MD process.

## 1. Introduction

In the 21st century, membrane distillation (MD) can be considered as the third-generation desalination technology. MD is a thermally driven membrane separation process that is applied for the separation of non-volatile solutes from aqueous solutions. In this process, porous hydrophobic membranes are required to retain the liquid phase in the feed side, whereas vapor gets transported through the microporous structure [1,2]. If the membrane is not sufficiently hydrophobic, membrane pores are easily filled with feed solution when they come in contact with the feed because of capillary forces. Currently, MD has been applied for more challenging wastewater streams with a higher load of corrosive contaminants compared with saline water desalination [3]. Using MD is advantageous as compared to other separation processes in terms of water flux and salt rejection. The prominent features of MD include (1) high rejection of ions and non-volatile solutes, (2) minimal requirement of operating pressure, (3) less demand of mechanical strength, (4) maximum recovery, and (5) less membrane fouling [4]. Reverse osmosis (RO) has been a widely used technique for seawater desalination. The ability to remove many dissolved substances/solutes efficiently is one of the salient features of the RO process. In addition to that, RO does not need any other chemicals as it separates dissolved substances from the feed stream. Apart from these prominent advantages, RO has several limitations as compared to MD process, which are as follows [5,6,7]: (1) Membrane fouling propensity is higher in RO process; whereas, lower membrane fouling occurs due to the reduced chemical interaction between membrane and process solution. (2) RO needs high operating pressure compared to MD process. (3) RO cannot be operated with high solute concentrations in the feed stream, or even with near-saturated solutions (unlike MD). (4) RO cannot work on low-grade heat, such as solar energy and geothermal energy, unlike MD. (5) RO requires high mechanical properties of the membrane, unlike MD. (6) Very importantly, unlike pressure-driven membrane processes (RO), MD does not need any pre-treatment of the feed stream or additives (e.g., acids or antiscalants) as the membranes are less sensitive to concentration polarization or membrane fouling [8].

The performance of MD depends upon two major factors: (i) surface and pore wetting, and (ii) fouling, due to the accumulation of various substances, including inorganic and organic (as well as biofilms), onto the membrane surface or inside the membrane pores. Separation in the MD process takes place by evaporation of certain substances in the feed side, transportation of the vapor through the membrane pores, and condensation on the permeate side. When the evaporated vapor does not pass through the membrane, but condenses inside the membrane, the membrane becomes wet. This wetting reduces the performance by blocking the passages of vapors, and when the pores filled with condensed liquid are connected from the feed side to the permeate side of the membrane, the feed solution passes through the membrane, and no further separation occurs. In addition, hydrophobic polymers may initiate hydrophobic–hydrophobic linking between membrane surface and foulants; hence, membrane pores that are passages for vapor are blocked because of fouling. Thus, wetting and fouling influences the selection of proper hydrophobic polymers for fabricating MD membranes, and there is a need for proper research and development for membrane modification to overcome the wetting and fouling issues.

**Figure 1:**
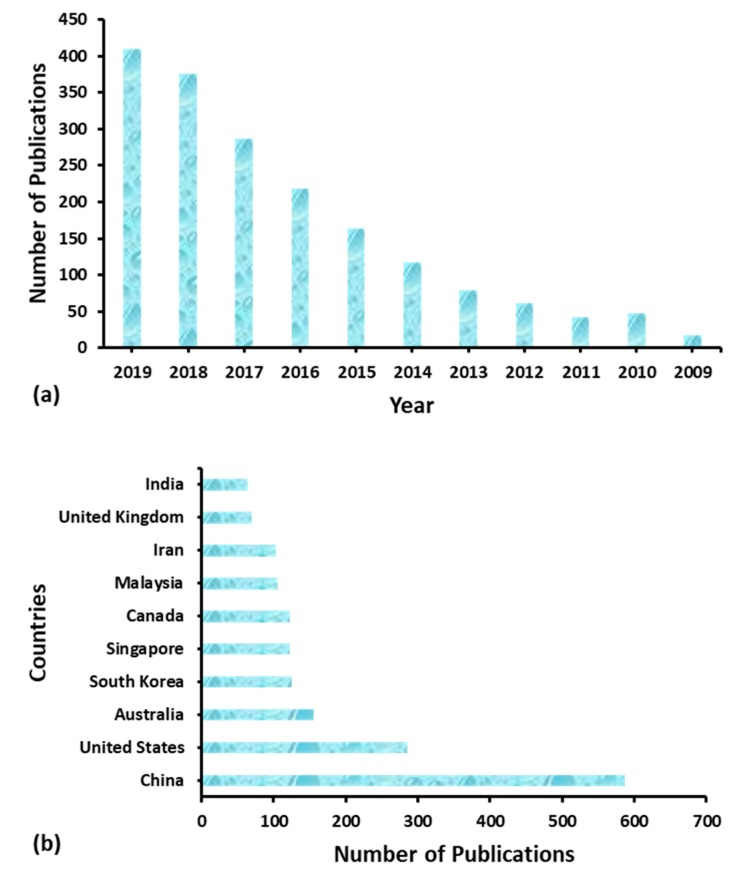
(**a**) Analysis of the number of published articles since 2009. Database obtained from the Advanced Scopus scholar search system with the terms “wetting” and “membrane distillation” as on August 2019. (**b**) Contribution of various countries to studies regarding membrane wetting in the membrane distillation (MD) process. Database obtained from the advanced Scopus scholar search system with the terms “wetting” and “membrane distillation” as on August 2019.

A thorough survey has been executed of peer-reviewed published articles related to “wetting” and “membrane distillation” over the last decade (Figure 1a). In addition, contributions to research and development based on wetting in MD from different countries are indicated in Figure 1b. The presented database has been acquired from the Scopus-based advanced scholar search system. After careful analysis of the database, it can be concluded that the wetting issue in the MD process has gained immense attention in the recent research and development of water treatment. Although, it requires more research to tackle the issue of membrane wetting in the MD process.

Nano and micro level hierarchical structures may cause an increase in the solid–liquid interfaces, thus, inducing traps of air bubbles within the membrane and affecting the improvement of antiwetting/antifouling characteristics of low energy surfaces. Despite the versatility and applicability of hierarchical structures and lower surface energy for MD membranes, a thorough blueprint for the preparation of such specialized membranes is rather less. To setup, a proper guideline for creating antiwetting superhydrophobic MD membranes, a demonstration of the factors that influence membrane hydrophobicity has been studied. Next, the effects of multilevel hierarchical structures and surface chemistry on superhydrophobicity of MD membranes were critically reviewed by demonstrating various feasible techniques for fabricating antiwetting surfaces.

## 2. Wetting of MD Membrane and Prevention of Wetting

**Figure 2:**
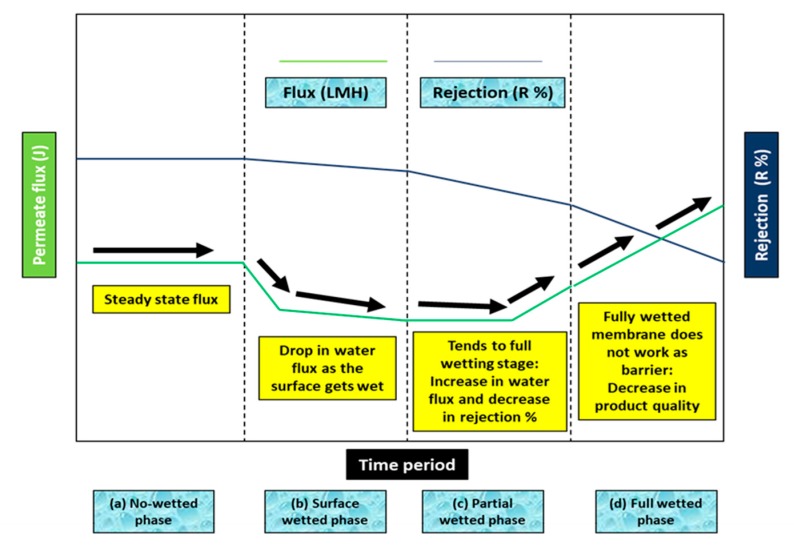
Degree of membrane pore wetting in the MD process: (**a**) No-wetting stage, (**b**) surface wetting stage, (**c**) partial wetting stage, (**d**) full wetting stage. [Note: LMH stands for L/hr/m^2^].

The occurrence of membrane pore wetting can decrease the performance of MD processes, and thus, wetting has become a prominent barrier to widespread, large-scale use of MD. When the membrane gets wet, the membrane starts losing its hydrophobic characteristics, leading to continuous water bridging. Membrane pore wetting can be categorized into four degrees: (1) non-wetted phase, (2) surface-wetted phase, (3) partially wetted phase, and (4) fully wetted phase [9]. The variation of membrane wetting degree has been indicated in Figure 2.

As indicated in Figure 2, after the non-wetted phase, the permeate flux gradually decreases because of the initiation of the surface wetting phase. Typically, the surface-wetted phase shifts the interface of the liquid/vapor inward of the cross-section of the MD membrane. Therefore, the feed stream penetrates the pore channels and eventually partial wetting of membrane occurs. The partial-wetted phase causes a reduction in permeate quality and may decrease the water permeate flux because of the minimization of the active surface area for mass transport. However, in some cases, it can cause an increase in permeate water flux because of wetting of some membrane pores. After long-term MD operation, full wetting takes place, where the membrane no longer acts as a barrier. This leads to a decrease in the rejection percentage [9].

Once the membrane gets wet, the MD process must be switched off to allow the membranes to dry. The membrane should be sometimes cleaned before drying because membrane fouling is closely associated with membrane wetting. The wetted MD membrane can be regenerated by the following methods: (1) drying of membrane, (2) air backwashing, and (3) chemical treatment. Table 1 indicates the advantages and overall outcomes of each technique to restore the wetted MD membrane. Some researchers have suggested that a slightly higher hydraulic pressure on the distillate side can be applied to prevent pore wetting when the area of the wetted membrane is not too large [10,11]. Currently, there is an increase in the number of studies regarding ultra-hydrophobicity, as well as antiwetting surfaces because of their potential self-cleaning ability.

**Figure 3:**
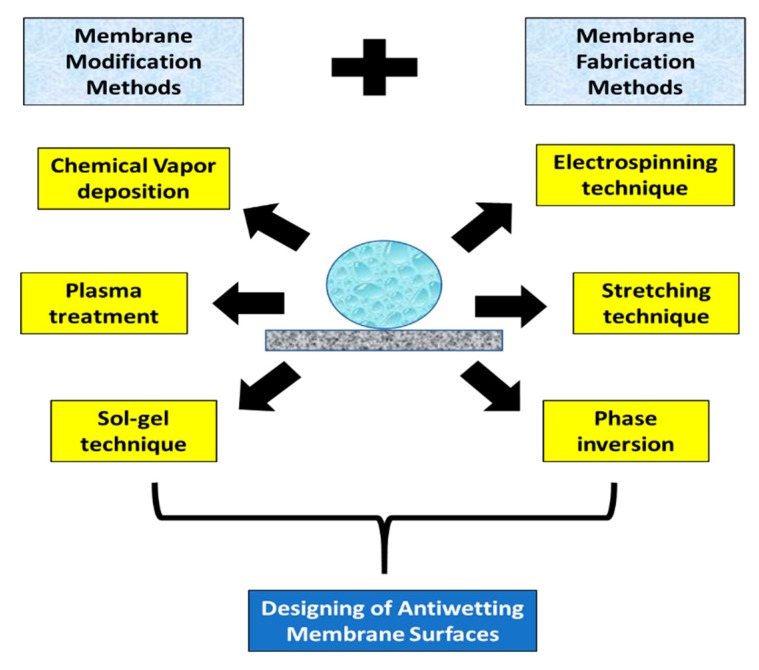
Feasible methodologies for designing antiwetting and superhydrophobic membrane surfaces.

Fundamentally, a water contact angle higher than 150° and a sliding angle lower than 20° indicate a superhydrophobic surface [15,16,17]. The surface wetting of the membrane can be reduced by two crucial methods, including modification of surface chemistry and enhancement of surface roughness. Optimization of operational conditions, such as pressure and temperature also can delay the wetting of the MD membranes. However, wetting and fouling of MD membranes are unavoidable. A diagrammatic representation of the feasible techniques for fabricating antiwetting surfaces is indicated in Figure 3.

Membrane pore wetting can be prevented by various methods which were suggested in much literature, and have been categorized into five major sections: (1) utilization of membrane adsorbents or laminations, (2) optimization of the operational conditions, (3) using composite membranes, (4) using asymmetric membranes, and (5) surface modification of polymeric membranes [10,18]. This review paper provides a tutorial and comprehensive perspective of MD membranes with insights toward better understanding its drawbacks. Moreover, the current developments in membrane modification by utilizing nanoparticles (NPs), as well as superhydrophobic additives to improve wetting and fouling resistance are discussed. As far as the research challenges related to the utilization of superhydrophobic membrane surfaces are concerned, the wetting resistance of superhydrophobic membranes fabricated by various techniques, durability, mechanical and chemical stability, as well as geometrical modeling to understand their wetting behavior, are all thoroughly reviewed in this paper.

## 3. Geometrical Models for Predicting Wetting Behavior

The water contact angle (CA) of a solid surface is an efficient way to measure the wettability of the surface. Hence, the hydrophobicity of a solid surface can be easily evaluated in terms of CA value. As far as wetting behavior of a membrane is concerned, it has been subdivided into two categories: (a) if the CA of the membrane surface corresponds to 90°–150°, it can be denoted as a hydrophobic surface; (b) if the CA of the membrane surface corresponds to >150°, it can be indicated as superhydrophobic surface. It is crucial to construct rational mathematical or theoretical models to demonstrate the wetting behavior mechanism and establish mathematical model equations to predict the wetting behavior of a solid surface [19,20].

### 3.1. Correlation of Contact Angle and Wetting of an Ideal Solid Surface

**Figure 4:**
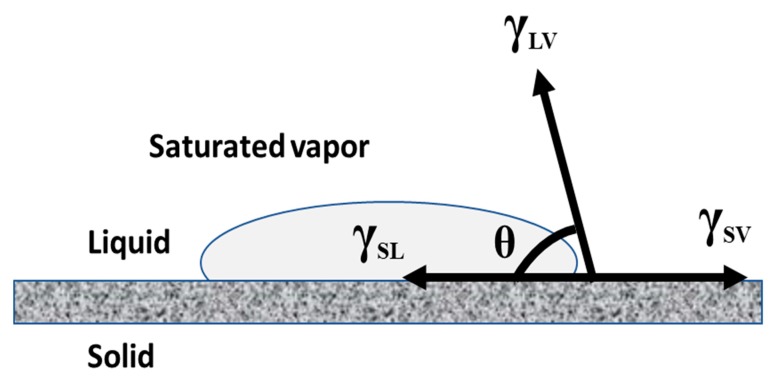
Diagrammatic representation of Young’s equilibrium contact angle (θ) of a solid surface, due to balancing of solid–liquid (γ_SL_), liquid-vapor (γ_LV_), and solid–vapor (γ_SV_) surface tensions.

Most liquids wet the solid surfaces and exhibit a CA of water. In 1805, Thomas Young described the CA of a liquid drop on an ideal solid surface which is defined by the mechanical equilibrium of the drop under the action of three interfacial tensions. Figure 4 indicates the equilibrium CA of a solid surface [21,22]. Equation (1) can be referred to as Young’s equation.

γ_SV_ = γ_SL_ + γ_LV_·Cosθ (1)

where γ_LV_, γ_SV_, and γ_SL_ indicate the liquid-vapor, solid–vapor, and solid–liquid interfacial tensions, respectively, and θ is the CA.

While considering Young’s equation for an ideal solid surface, the influences of surface roughness, chemical heterogeneity, swelling, and surface reconstruction are typically neglected. In general, complete wetting takes place when θ = 0 and typically occurs for liquids with lower surface tension *γ*_LV_ and on solids with high surface energy *γ*_SV_. When *γ*_SV_ > *γ*_SL_, then 0° < θ < 90°, and when *γ*_SL_ > *γ*_SV_, then 90° < θ < 180°. As per Young’s equation, it is evident that the interfacial tension between the solid and liquid *γ*_SL_ is lower than *γ*_SV_ only when θ < 90°; this occurs in the case of wetting. In contrast, *γ*_SL_ > *γ*_SV_ can occur only when θ > 90°; the area of contact of the liquid–solid interface will be minimized. In this case, the liquid behaves in a non-wetting manner because *γ*_LV_ is always finite and positive. Then, the non-wetting behavior reduces the total surface/interfacial energy of the liquid. Therefore, the water CA depends on the optimization of the contact area of the solid–liquid interface and liquid–air interface. Importantly, the wetting behavior of a liquid on an ideal solid substrate can also be demonstrated from the surface thermodynamics by analyzing the work of adhesion, thus, leading to Young’s equation [23,24,25]. Table 2 indicates co-relations between surface tension and the wetting of a solid.

Although these techniques for evaluating CA have been developed, these are not accurate enough to evaluate the wetting state of the membrane surfaces. CA analysis is a technique that uses image processing methodology, but it is prone to partial inaccuracies [26]. Typically, while analyzing using optical devices, the area covered by the droplet close to the three-phase contact line is either blurred or distorted, due to optical errors, resulting in substantial inaccuracies, while identifying the tangent line and droplet shape [27]. The errors in droplet shape and tangent line depend upon the resolution of the image. As per a previous report, it has been shown that an error of 1 μm in a baseline location may result in an inaccuracy of 10° in CA measurement [28].

Eventually, the CA analysis has been adjudged as the key criterion for the evaluation of superhydrophobicity of the membrane surface, but it cannot act as the only tool for evaluating the antiwetting behavior. Thus, sliding angle analysis has been relatively developed. The superhydrophobic membranes with lower sliding angle demonstrate antiwetting and self-cleaning properties, where water droplets roll off with dirt and foulants. In contrast, the superhydrophobic membranes with higher sliding angle may not show the self-cleaning effect because of the high dragging of fluid flow [29]. Interestingly, some researchers have observed that a membrane surface with a high CA does not always have a lower sliding angle, which depends on the weight of the water droplet when it begins to roll-off onto the inclined plate [30]. An exceptional example can be cited based on the observation of Murase et al., who indicated that a fluoropolymer with a CA of 117° shows a higher sliding angle than a poly-dimethylsiloxane with the CA of 102° [31].

**Figure 5:**
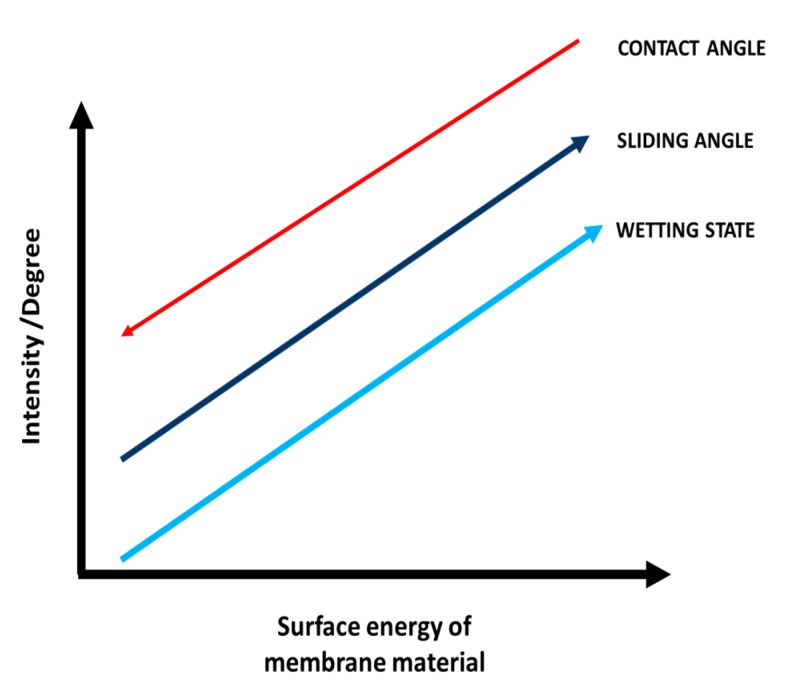
Correlations between wetting state, contact angle and surface energy of the material.

It is important to establish a correlation between CA, sliding angle, surface energy, and wetting. To understand membrane wetting, it is necessary to understand the interplay between the dynamics of spreading and the surface energy components of solids and liquids. Typically, wetting can be defined as the interaction between a liquid that comes in contact with a solid surface in the air [32]. A diagrammatic representation of the correlations between wetting state, CA, and wetting state as a function of the total surface energy is indicated in Figure 5, which indicates that lower spreading values demonstrate higher liquid repelling abilities, i.e., superhydrophobic behavior.

### 3.2. Wetting State of a Real Solid Surface

Typically, in order to demonstrate the water CA on a real solid surface, two models have been adopted—the Wenzel model and the Cassie–Baxter model. These models contradict the ideal solid surface because a real solid surface may possess surface roughness and chemical heterogeneity. Surface roughness with chemical homogeneity is considered in the Wenzel model, whereas chemical heterogeneity with a flat surface is considered in the Cassie–Baxter model. The surface roughness r in the Wenzel model is defined as the ratio of the actual area to the projected area of the surface [33,34,35]. Mathematically, the Wenzel equation is given as Equation (2):

Cos θ* = r Cos θ_Y_ (2)

where θ* indicates the apparent water CA and θ_Y_ is the equilibrium water CA from Young’s equation on an ideal solid surface without considering surface roughness. According to the Cassie–Baxter model, f_1_ represents the area fraction of the solid, whereas f_2_ represents the area fraction of air under a drop on the substrate. Mathematically, the Cassie–Baxter equation can be indicated, as mentioned in Equation (3) [36,37,38]:

Cos θ* = f_1_ Cos θ_Y_ + f_2_ Cos θ_Y_’ (3)

where θ* indicates the apparent water CA, θ_Y_ is the equilibrium water CA on the solid, and θ_Y_’ is equivalent to 180°.

**Figure 6:**
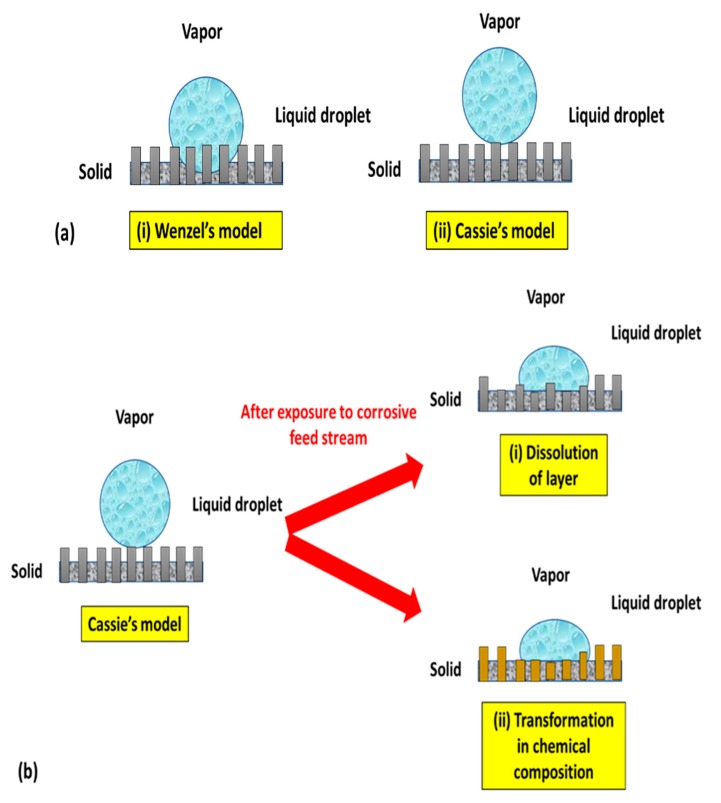
(**a**) Model of the wetting phenomenon: (i) Wenzel’s model and (ii) Cassie’s model. (**b**) Mechanism showing chemical instability and change in Cassie’s model to Wenzel’s model because of (i) dissolution of the layer and (ii) chemical transformation.

Typically, the surface wetting follows either the Cassie–Baxter wetting model or the Wenzel wetting model. In the Cassie–Baxter state, air trapped in the grooves between surface features forms a composite (air/solid) hydrophobic surface, leading to a larger contact angle compared to the contact angle θ with a flat surface. In contrast, in the Wenzel state, the liquid on the surface enters the grooves, leading to higher surface wettability, due to the increase in contact area [7,39,40]. Therefore, it can be concluded from the Wenzel equation that surface roughness amplifies the wettability of the original surface. In other words, a hydrophilic surface becomes more hydrophilic, whereas a hydrophobic surface is more hydrophobic in nature. The area fraction under the water drop in the Cassie–Baxter model is crucial such that the larger the area fraction of air under a drop on the substrate, the higher the water CA [34,41]. Even though the Wenzel and Cassie–Baxter mathematical models were proposed long ago, these models have been widely utilized to develop antiwetting or superhydrophobic surfaces [27,42]. Figure 6a provides a pictorial representation of the Wenzel and Cassie–Baxter models. However, in some cases, the superhydrophobic coatings onto the MD membrane seem to be unstable because of poor adhesion or non-uniform distribution of nanoparticles onto the membrane macrostructure. Figure 6b indicates the chemical instability of the surface layer, due to the dissolution of the layer, as well as a change in the chemical composition. This state also shows the transformation of the Cassie–Baxter model to the Wenzel state.

## 4. Science and Engineering of Antiwetting Interfaces

In this section, fabrication techniques for antiwetting surfaces for MD application are reviewed. An important method to minimize surface wetting is to utilize superhydrophobic materials or coatings to coat the polymeric membrane. Typically, a superhydrophobic layer creates a layer of air between the feed stream and the surface, and thus, this specialized superhydrophobic surface prevents contact between the surface and the feed stream m [43,44,45]. These antiwetting surfaces minimize the area of real contact between the membrane surface and feed stream, thus, maximizing wetting resistance. However, unfortunately, after long-term operation, the superhydrophobic surfaces may start to lose their antiwetting properties once exposed to the feed stream. Therefore, a proper approach to enhance stability in long-term operation is required. In addition, in some cases, unavoidable abrading forces cause the wetting model to be transformed from the Cassie–Baxter model to the Wenzel model, thereby enabling the liquid and solutes to penetrate the membrane pores and destroying the superhydrophobic property [46,47]. Thus, durability or mechanical stability is an important factor that leads to a reduction in antiwetting features. The MD membranes must satisfy certain requirements enlisted in Table 3 with appropriate conditions to ensure high permeability with minimized wetting in the MD process. Before delving into the details of fabricating antiwetting surfaces, the basic fundamentals and factors affecting membrane wetting are discussed.

### 4.1. Basics of Wetting Issue in MD

Typically, the performance of MDs fails because of the following reasons: (a) a decline in water flux, due to membrane fouling (external and internal fouling) and (b) membrane and pore wetting, where the liquid penetrates through the membrane pores and deteriorates the quality of the permeate [55,56,57]. Membrane wetting is considered a serious issue because it contaminates the permeate. In addition, fouling may accelerate membrane wetting, thus, reducing vapor flux. Therefore, fouling and wetting are interrelated in the MD process. Membrane wetting leads to increased membrane fouling, and interestingly, membrane fouling results in the increased membrane and pore wetting. Table 4 indicates the prominent causes of MD membrane wetting. Inorganic foulants may lead to partial or full wetting because of pore blocking or pore clogging of inorganic crystals, which reduce permeate quality and damage pore structures [57]. In contrast, organic foulants lead to predominant surface wetting because of absorption of organic matter onto the membrane surface and pores, which reduces permeate quality in terms of TOC [58]. In addition, MD membrane wetting can occur because of continuous chemical degradation of the polymeric membrane, thus, forming hydrophilic chemical groups on the membrane surface. Furthermore, prolonged use of MD membrane may lead to loss of membrane hydrophobicity, resulting in deterioration of the chemical and mechanical stability which makes MD operation difficult. Moreover, membrane wetting also depends upon the composition of the feed stream. Typically, the surface tension of seawater solutions is higher than that of distilled water (72 mN·m^−1^). Thus, the possibility of wetting membrane pores and surfaces in low when seawater solutions are used. However, when the feed stream consists of organic solutes or surfactants, the surface tension steeply decreases. Importantly, if the surfactant concentration is higher than a critical value, membrane wetting will occur [59].

As mentioned in Table 4, membrane wetting can also be demonstrated in terms of liquid entry pressure (LEP). LEP can be defined as the minimum transmembrane pressure required for the feed solution to penetrate through the membrane pores. The LEP value should be as high as possible. This condition can be achieved by increasing the average CA and decreasing the pore size range [63,64]. Mathematically, LEP can be expressed as indicated in Equation (4) [65]:

(4) $$\mathrm{LEP}=\frac{-2\gamma_{L}\;\cos\theta }{r_{\max}}$$

where γ_L_ indicates the surface tension of the feed stream, θ represents the water CA, and *r*_max_ the maximal membrane pore radius.

### 4.2. Factors Affecting Membrane Wetting

In this section, the factors affecting membrane wetting are reviewed. Basically, operational conditions, membrane properties, and solution chemistry are the main factors that influence membrane wetting, as well as fouling in the MD process. Flowrate and operating pressure are most prominent operational conditions, whereas membrane properties, such as thickness, pore size distribution, porosity, and surface energy may influence the wetting and fouling in the MD process [66,67]. Temperature, pH, and presence of dissolved gases, are typical factors of wetting based on solution chemistry. As per the dynamics of the MD process, the system must meet the following criteria [68]. Table 5 demonstrates the prominent criteria for improved MD performance in terms of wetting and fouling resistance conditions.

### 4.3. Feasible Techniques to Fabricate Antiwetting Surfaces

**Figure 7:**
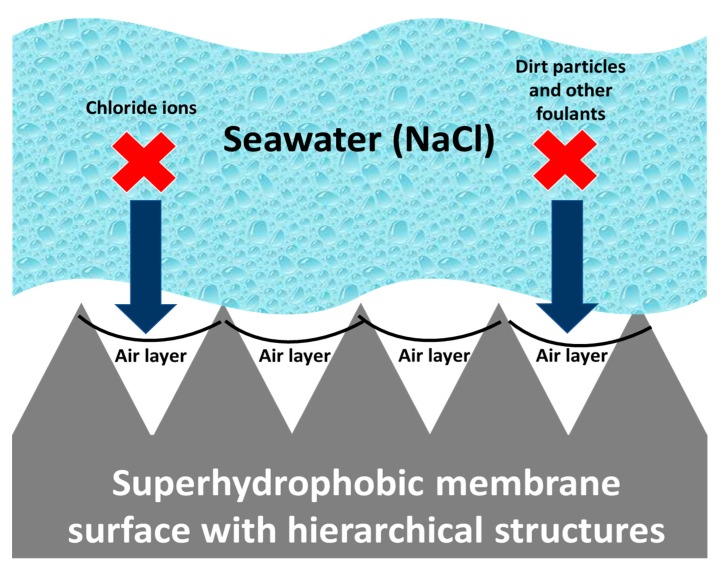
Antifouling mechanism shown by superhydrophobic membrane surface with hierarchical structures.

Typically, the fabrication of antiwetting and superhydrophobic surfaces requires surface roughening at the micro or nanostructure level followed by a surface modification which would result in lower surface energy. Some methodologies, such as deep coating, chemical etching, spray coating, and solution-immersion utilize various coating agents for surface modification of the MD membrane after roughening of the surface, whereas other techniques, such as laser electrodeposition and template deposition do not require surface modification [77,78,79]. A facile, economical, less time consuming, and mechanically stable approach is crucial for the casting process. In addition, features, such as chemical stability, fouling resistance, and durability of the superhydrophobic surfaces have been achieved via various techniques. For desalination in the MD process, the basic mechanism for fabricating antifouling and antiwetting surfaces involves the formation of an air layer onto the surface. This prevents corrosive ions, such as chloride, dirt particles, and other foulants from invading the membrane surface. This can be explained based on hierarchical structures that can easily trap a considerable amount of air within the valleys between the roughened membrane surface when an ultrahydrophobic surface comes in contact with foulants [80]. This above-mentioned phenomenon has been diagrammatically explained in Figure 7.

In this section, membrane fabrication techniques, such as electrospinning, phase inversion, and mechanical stretching are critically reviewed to understand the basic fundamentals for fabricating antiwetting surfaces for MD application. Moreover, membrane modification methodologies, such as blending or coating, plasma treatment, and chemical vapor deposition have also been demonstrated, which are generally combined with the above-mentioned fabrication techniques to produce superhydrophobic membrane surfaces. The commonly used techniques have been categorized into two parts—(a) wet chemical processes and (b) dry physical processes. Under wet chemical processes, various techniques, such as sol–gel process, electrospinning, and phase inversion have been elaborated in this section. The state-of-art methodologies for each technique have been presented. Dry physical processes typically include heating, extruding, annealing, and stretching. For example, plasma treatment for membrane modification can be cited as an example of the dry physical process. In this section, membrane fabrication techniques for producing MD membranes have been explored, and then, various membrane modification techniques to fabricate specialized antiwetting membrane surfaces with hierarchical nano/microstructures or lower surface energy are indicated.

#### 4.3.1. Feasible Membrane Fabrication Techniques for MD Membranes

In this section, various feasible membrane fabrications methodologies have been thoroughly reviewed in terms of advancements, and novel case studies applied with different membrane surface modifications are discussed. Electrospinning, phase inversion, and mechanical stretching methods are described with high scientific discussions for casting hydrophobic and antiwetting MD membranes.

##### Electrospinning Technique for Membrane Fabrication

**Figure 8:**
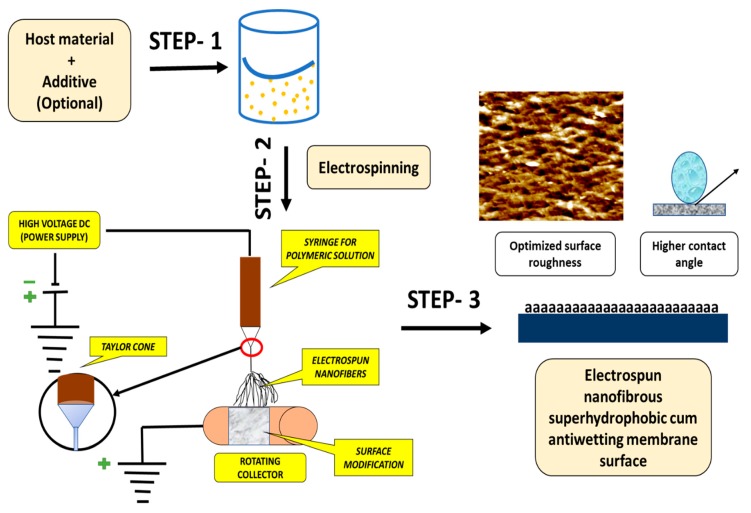
A schematic diagram showing the process involved in the fabrication of superhydrophobic membrane surfaces via electrospinning.

Electrospinning is an efficient method for fabricating non-woven nanofibrous layers with high surface roughness and high porosity. Basically, electrospinning set-up consists of three major components: (1) a high-voltage supplier, (2) a syringe/capillary tube with needles, and (3) a metal collecting roller. In this technique, a high voltage is used to create an electrically charged jet of the polymer solution, so that, just before reaching the collecting roller, the solution jet evaporates and simultaneously solidifies, and then collected as an interconnected web of small fibers [81]. Typically, electrospinning is a versatile method for fabrication of constant nanofibrous with high surface bumpiness which leads to increased hydrophobicity. Even superhydrophobic membranes were fabricated by lowering the surface energy of the composite membrane [82]. The polymer structures fabricated by electrospinning offer a high surface area to pore volume ratio, a high surface area, and good mechanical stability. Superhydrophobic polymer-based siloxane mats can be fabricated via electrospinning which can be further used in the MD process for enhanced performance in terms of water flux and salt rejection [83,84]. However, to utilize these membranes on a larger scale, membrane pore size, porosity, surface roughness, and SiO_2_ composition must be optimized for long-term MD performance. A schematic flow chart diagram of electrospun membranes is shown in Figure 8.

Tijing et al. prepared a novel electrospun nanofibrous membrane with nanofillers for MD application. In this study, a superhydrophobic electrospun nanofibrous membrane was cased using polyvinylidene fluoride-co-hexafluoropropylene (PcH). In addition, carbon nanotubes (CNTs) were utilized in different concentrations as nanofillers to improve the hydrophobic and mechanical properties of the membrane. The dual-layer concept was applied using two cohesive layers comprising a thin CNT-PcH top active layer and a thick PcH supportive bottom layer. Interestingly, the overall outcomes demonstrate that the water CA increases up to 158° after doping with CNTs because of increased surface roughness [63].

Khayet et al. modified the surface of a PSF nanofibrous layer by surface segregation. In this study, 6 wt % fluorinated polyurethane additive (FPA) was added to the polysulfone (PSF) blend via electrospinning. During the electrospinning process, FPA underwent spontaneous surface segregation leading to specialized nanofibers with enhanced superhydrophobicity because of the presence of a fluorine-rich surface. These electrospun nanofibrous membranes were tested for desalination application in the MD process, and a stable water flux was achieved [85]. Dong et al. demonstrated that a ultrahydrophobic composite membrane could be fabricated by combining an electrospun PVDF and PTFE nanofibrous scaffold doped with a microporous PTFE micro-powder. Interestingly, it was found that the CA changes from 130° to 151° by changing the PTFE micro-powder concentration in the dope solutions from 0 wt % to 12 wt %. A CA of 151° was observed because of the micro and nano level hierarchical structures onto the membrane surface [86]. Furthermore, Su et al. obtained similar results based on a combination of electrospraying and electrospinning, wherein a porous membrane with superhydrophobic features was cast through simultaneous electrospraying of silica/DMAc colloids and electrospinning of PVDF/DMAc solutions. The novel hybrid membrane with micro and nano hierarchical structures showed an increased CA of 160° and a lower sliding angle of 3°. In addition, this novel hybrid membrane showed excellent membrane performance in MD operation [87].

A robust superhydrophobic electrospun nanofibrous membrane can only be designed on the basis of the three criteria in Table 6. In addition, there are several important factors that must be considered, while fabricating antiwetting electrospun nanofibrous surfaces: (a) A combination of lower surface energy and hierarchical structure is equally important; (b) hydrophobic treatment of the polymeric nanofibrous mats must be consistent, and uniform; and (c) the interconnected pore structures should not be damaged during application [77,88].

Although there are numerous advantages of electrospinning for the fabrication of superhydrophobic antiwetting surfaces, there are certain limitations where more research and development is required:

1. The longevity of electrospun surfaces requires some improvement to prevent observed flaking or pops after the heat transfer tests [92].
2. The electrospun polymer coatings onto the desired substrates possess a higher thickness, and this results in higher mass transfer resistance [93].

##### Phase Inversion Membrane Casting Technique

**Figure 9:**
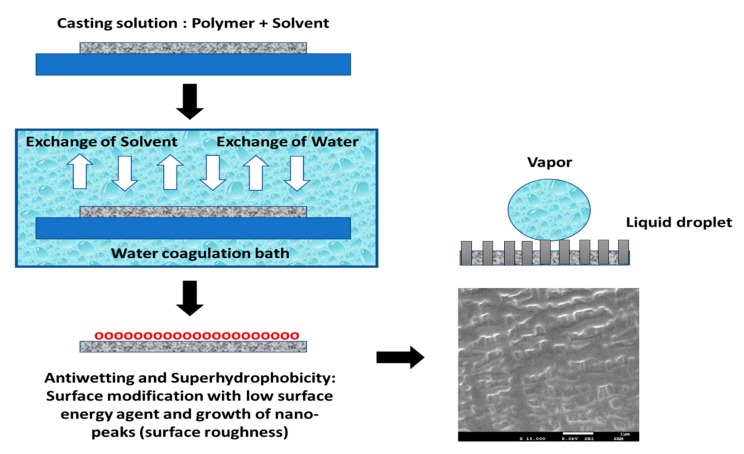
Illustration of the phase inversion method for casting self-cleaning surfaces either by surface modification with low surface energy or enhancement of surface roughness.

The mechanism and state-of-art of phase inversion technique are reviewed for producing antiwetting and superhydrophobic MD membrane surfaces. Typically, phase inversion is a de-mixing process where a homogeneous polymeric solution phase is changed into a solid phase under optimum conditions. The process of membrane fabrication by phase inversion has been demonstrated in three steps [94,95]:

1. First, polymer pellets are dissolved in a particular organic solvent to form a polymeric casting solution, which is then cast on a flat plate up to the desired thickness with a knife casting device.
2. Next, the semi-liquid film is cast onto the plate and is allowed to be immersed in a nonsolvent bath for precipitation.
3. Finally, a polymeric film is rapidly formed with an asymmetric structure because of the exchange of solvent and nonsolvent across the interface, which can be explained based on the absorption of water molecules by the polymeric substrate and simultaneous loss of solvent. Figure 9 indicates the phase inversion concept utilized for fabricating self-cleaning membrane surfaces for MD application.

Before selecting polymers for fabrication of superhydrophobic membranes via phase inversion, the materials must fulfill the following criteria: (1) highly hydrophobic polymer materials should be selected, (2) the materials should be easily available, and fabrication and assembly should be simple, (3) the feed stream liquid and polymeric membrane surface must be compatible, and (4) operating conditions must be optimized [96,97].

Munirasu et al. have fabricated a superhydrophobic PVDF membrane via the phase inversion technique using alcohols, such as ethanol and methanol as nonsolvent on non-woven support. In this study, subsequent mechanical scratching was applied to induce superhydrophobic behavior of the whole membrane surface. The superhydrophobic features of the PVDF membrane are due to the sponge-like interconnected fibrous microstructure [98]. Ray et al. fabricated a newly designed antiwetting membrane surface via phase inversion composed of octadecyltrimethoxysilane (OTMS) by doping carbon black (CB) into a PVDF solution. OTMSs were analyzed to be an ideal agent for increasing the superhydrophobic features of the PVDF membrane. The composite membrane was found to be ultrahydrophobic with a CA of more than 150° and showed high membrane performance in terms of salt rejection and water flux. In addition, the casted membrane was reused after physical cleaning to evaluate wetting resistance [44]. In another interesting study, Wu et al. developed a superhydrophobic membrane where the PVDF solution was doped with hydroxyl rich silica particles via phase inversion. The increased CA is due to the improved surface roughness, where silica particles acted as the nuclei for crystallization. Additionally, the 3-D growth of polymer-based spherulites was due to the hydrogen bond created between the hydroxyl groups and PVDF polymeric chains. The water flux increased to 2.7 times that of the virgin membranes in the MD process [99]. Interestingly, Xiao et al. demonstrated the combined effect of phase inversion and plasma treatment resulting in a superhydrophobic membrane surface. A new hybrid superhydrophobic PVDF membrane with micro-pillar arrays (MP-PVDF) using a micro-molding phase separation (μPS) method was thoroughly investigated. Consequently, the fabricated membrane indicated a CA of 166° and a sliding angle of 15°. However, after CF_4_ plasma treatment, the modified superhydrophobic membrane indicated a lower sliding angle of 3.0°. Furthermore, the fabricated membrane showed less scaling and fouling during long-term operation in the MD process [100].

Typically, the phase inversion technique can be applied to fabricate flat sheet membranes, as well as hollow fiber membranes. For instance, flat sheet membranes can be fabricated when a polymeric solution is cast onto a flat glass, and is then immersed in a precipitation bath. The structural characteristics of the pores of the fabricated flat sheet membrane depend on the rate of exchange of the organic solvent and non-solvent (e.g., water) [101]. In contrast, hollow fiber membranes fabricated via phase inversion involve the extrusion of the polymeric solution, coagulation, and sintering of the precipitated hollow fiber. Based on superhydrophobic linear low-density polyethylene (LLDPE), Yuan et al. prepared and eventually analyzed antiwetting features and mechanical stability [102]. Initially, polymeric pellets were dissolved in xylene and cast onto a flat glass substrate, where it was kept to dry. After maintaining at 120 °C, a consistent and uniform LLDPE membrane surface was obtained with a CA of 102°. Eventually, to enhance the CA, the roughness of the membrane surface was increased by drying at a temperature of 5–10 °C. Typically, with regard to surface chemistry, slow evaporation of the solvent occurs because of low temperature, thus, resulting in phase inversion and forming pores onto the surface of LLPDE. Interestingly, the CA increased to 127°. However, to form a superhydrophobic surface, the membrane surface roughness needs to be increased by applying ethanol to the LLDPE solution. In this process, ethanol acts as a precipitator and aggregates some portion of LLPDE, which again act as nuclei for further LLPDE aggregation. This leads to further phase inversion and an increase in membrane surface roughness. Thus, a CA of 153° and a sliding angle of 10° can be achieved. This indicates the property of the self-cleaning surface, and the membrane was found to be mechanically and chemically stable.

##### Mechanical Stretching for Hollow Fiber Membrane Fabrication

**Figure 10:**
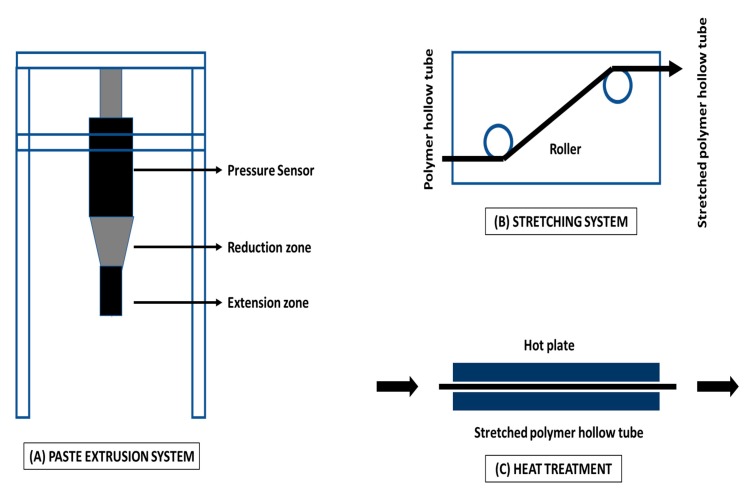
Flowchart diagram demonstrating the fabrication of hydrophobic polymeric hollow fiber membranes for MD application.

The stretching technique is appropriate for crystalline polymers, such as PP, PTFE, and PE. Typically, stretching of membranes can be considered one of the cheapest methods. In this technique, the polymer is generally stretched either uniaxially or biaxially in the direction of the extrusion to produce an extruded foil or membrane. As a result, the crystalline portions are located parallel to the extrusion direction. The polymeric membrane causes micro ruptures because of the exerted mechanical stress onto the polymeric membrane [96]. Therefore, a porous polymeric membrane is produced with a pore size in the range of 0.1 µm to 1 µm. Many new studies have revealed that membrane stretching can be utilized as an alternative to enhance membrane pore size, physical and surface chemistry, and porosity. There are some crucial parameters that must be considered: (a) type of stretching mode (whether uniaxial or biaxial), (b) tensile force of stretching, (c) rate of stretching, and (d) heat treatment. In general, optimal heat treatment is applied during membrane stretching, which can ensure the porous structure of the membrane and can stabilize the mechanical properties of the fabricated membrane. Likewise, Wang et al. [103] prepared a polymeric hollow fiber membrane by utilizing paste extrusion and stretching, followed by heat pressing treatment. A typical flowchart diagram has been presented in Figure 10 to demonstrate the fabrication of hydrophobic polymeric hollow fiber membrane by stretching.

Typically, hollow fiber MD membranes are cast by the mechanical stretching technique. Mechanical stretching is not only a difficult process, but also cannot fabricate membrane surfaces with a narrow pore size and high porosity. Controlling the operational parameters is crucial to cast membranes with high permeability. Kurumada et al. demonstrated changes in pore structure after uniaxial and biaxial mechanical stretching of the membrane and interestingly observed that uniaxial stretching resulted in island-like structures, whereas biaxial stretching resulted in lattice-like porous structures [104]. In other studies, asymmetric heat treatment during mechanical stretching improved the porosity of the membrane and minimized the pore size range [105]. Another example of surface modification for achieving superhydrophobicity is the fabrication of membranes via extrusion. Huang et al. reported unique structures fabricated from a mixture of a PTFE emulsion and a PVA aqueous solution. Micro and nanostructures were created by PTFE crystallization on PTFE membranes, which showed superhydrophobic characteristics with a CA of 155° and a sliding angle of 8°. During this process, the micro level structures aggregates forming the crystalline phase, whereas the nano level structures created the amorphous phase. Interestingly, water flux in the VMD process improved with the enhancement in average pore size when the PTFE membrane was subjected to stretching [106]. Earlier, Fang et al. reported the fabrication of highly hydrophobic porous alumina ceramic hollow fiber membranes via extrusion and sintering for MD application. The authors transformed the surface chemistry from hydrophilic to hydrophobic by grafting fluoroalkylsilane onto the surface of hollow fiber membranes. The average pore size was found to be 0.7 µm, which is comparable to a commercial membrane. The fabricated hollow fiber membranes were applied in the VMD process to analyze the performance. Interestingly, it was observed that the salt rejection and water flux were recovered after continuous water washing and drying [107].

#### 4.3.2. Membrane Surface Modification Methodologies

To achieve superhydrophobicity and antiwetting membrane surfaces, membrane modification is an essential step. Blending or coating, commonly called sol–gel processing, is considered the most feasible method for enhancing the hydrophobicity of MD membranes using various additives, which form a 3-D sol–gel network system onto the membrane. Apart from this, plasma treatment and chemical vapor deposition techniques are also explored for MD membrane modification. Furthermore, few novel case studies have applied various membrane fabrication techniques, which are discussed here.

##### Blending or Coating for Antiwetting Membrane Surfaces

**Figure 11:**
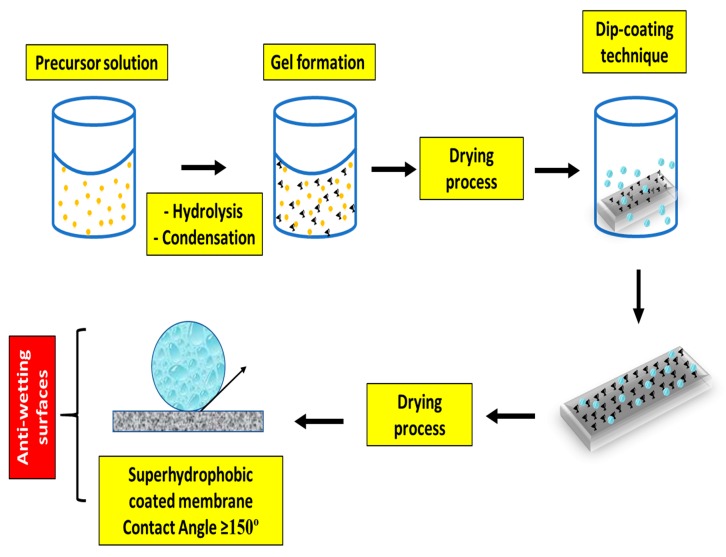
Schematic presentation of antiwetting and superhydrophobic coatings of MD membrane by sol–gel processing technique.

Blending or coating has been extensively applied for fabrication of antiwetting and superhydrophobic membranes because it can provide a rough surface for the membrane. This technique may involve inorganic silica and organically-modified silanes, which create a 3-D sol–gel network system [108]. By incorporating organically-modified or functionalized silanes or inorganic silica nanoparticles, wetting resistance, good mechanical properties, and good adhesion can be easily achieved. In general, adhesive properties can be achieved by forming a covalent bond between the surface coatings and the substrate under optimal conditions. Additionally, organic components as precursors may help achieve the desired membrane surface roughness for enhancing the superhydrophobicity [109]. Figure 11 indicates a thorough protocol for fabricating superhydrophobic MD membranes involved in the sol–gel process, followed by surface coating.

Superhydrophobic and antiwetting surfaces were fabricated by doping organic fluoroalkyl silane in hybrid sol–gel matrix consisting of silica nanoparticles [110]. However, sometimes the CA fails to achieve 150° in the case of flat and smooth surface coated with fluoroalkyl silane. In addition, fluoroalkyl silane is more expensive than other required materials. Hence, the surface roughness produced by silica nanoparticles has a key role in the fabrication of antiwetting and superhydrophobic MD membranes [111]. Recently, Ray et al. demonstrated the successful fabrication of a triple-layered MD membrane consisting of a top ultrahydrophobic layer onto a polypropylene mat via sol–gel processing of octadecyltrimethoxysilane (OTMS), and the bottom layer was fabricated with a hydrophilic poly(vinyl alcohol) polymer via the phase inversion technique. Interestingly, the triple-layered membrane exhibited superhydrophobicity, with an average CA of more than 150°, and showed high membrane performance in terms of permeability and salt rejection [64]. Similarly, Wang et al. modified polypropylene membranes by embedding SiO_2_ nanoparticles along with 1H,1H,2H,2H-perfluorodecyltriethoxysilane, which showed lower surface energy. In this study, SiO_2_ nanoparticles were deposited via in-situ sol–gel processing to form a rough surface onto the PP membrane. The overall results demonstrated excellent antiwetting and antifouling performance in the VMD process to treat high strength seawater. Furthermore, the fouling rate decreased to 20% in the case of superhydrophobic composite membrane compared with that of the original membrane [112]. The coating technique has been applied to fabricate electrospun nanofibrous membrane for producing superhydrophobic MD membranes. For example, Dizge et al. cast a superhydrophobic membrane by growing SiNPs onto cellulosic fibers by utilizing an in-situ sol–gel processing technique. Eventually, the SiNPs grafted onto the cellulosic nanofiber were coated with fluoroalkylsilane to lower the surface energy of the composite membrane. The fabricated membrane showed high performance in terms of wetting resistance and indicated stable water flux in MD operation of 10 hours [112]. Similarly, Liao et al. fabricated a dual layered superhydrophobic membrane consisting of PVDF incorporated with silica nanoparticles for desalination in the MD process [90,113]. Interestingly, these membranes performed much better in terms of water flux, as well as rejection rates compared with the virgin electrospun nanofibrous membranes. The incorporation of certain nanoparticles or silane-modified/coated nanoparticles transforms the hydrophobic membranes to superhydrophobic ones; hence, it helps in the prevention of membrane wetting and fouling in the MD process. A brief summary of nanoparticle incorporated membranes for MD application is demonstrated in Table 7.

In the MD process, high permeation without surface or pore wetting, proper pore size range (with high salt rejection) and with tortuosity close to one (cylindrical) is highly desired [123]. Due to this, nanofillers based membrane gained much attention for their high functionality and selectivity. For example, carbon nanotubes (CNTs) are considered to be one of the most promising nanomaterials/nanofillers for membrane fabrication because they can modify the membrane’s architecture to achieve efficient physical/chemical properties [124,125,126]. In other words, it can also be regarded as mixed matrix membranes (MMMs). In general, MMMs are a new-generation membrane for water and gas purification applications and became an emerging composite material for research and development in both academic and industrial interests because of unique properties of inorganic fillers. In addition to that, this concept is based on high selectivity of the dispersed fillers, desirable mechanical properties, and economical processability of polymers [127,128]. Typically, the inorganic nanofillers are found to be porous molecular sieve-type materials, which includes carbon molecular sieves and zeolites, etc. Recent literature has reported the development of a novel type of MMMs hydrophobic hollow fiber membrane, as well as superhydrophobic flat sheet membrane with nanosized pores to improve the desalination performance in the MD process [129,130].

Spray-deposition can be considered a simple process designed for industrial coatings. The spray-deposition technique has been used to fabricate antiwetting and superhydrophobic surfaces by optimizing the concentration of the precursor and spraying conditions to achieve a binary micro/nanostructure [131]. Zhang et al. produced antiwetting and superhydrophobic hollow fiber membranes by spray-deposition for MD application. Interestingly, antiwetting effects and a higher mass transfer flux were achieved by these fabricated hollow fiber superhydrophobic membranes [108,132]. Thus, the spray deposition technique can be followed minutely for further research and developments of antiwetting and superhydrophobic surfaces.

##### Plasma Treatment for Membrane Surface Modification

**Figure 12:**
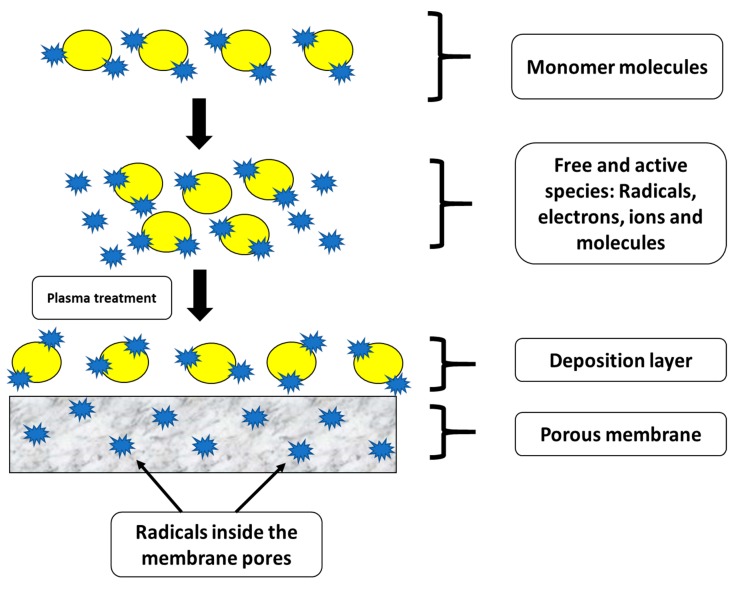
Mechanism involved in the physical and chemical interactions during plasma polymerization.

Plasma treatment is a dry physical process that involves physical, as well as chemical interaction of the active species onto the polymeric surface. Typically, plasma treatment involves post-treatment for surface modification of the polymeric membrane to achieve the desired characteristics without transforming the membrane matrix or composition of the membrane. This process consists of adsorption and polymerization of the ionized gas on the membrane surface [133]. Figure 12 indicates the mechanism involved in plasma polymerization for thin-film deposition. During plasma treatment, initially, a target monomer is evaporated and pumped into a chamber under vacuum conditions. Then, a lamp is used to ionize the monomer molecules, resulting in the cleavage of monomer molecules to generate free and active electrons, ions, molecules, and radicals. These free and active electrons, ions, molecules, and radicals are adsorbed, condensed, and polymerized onto the surface of the membranes. The free and active electrons, as well as ions, undergo cross-linking among the deposited molecules, which slowly harden to create a dense coating layer. However, there is a high possibility for the free and active electrons, ions, molecules, and radicals to penetrate into the polymeric chains through the pores of the membrane. Thus, the polymeric membrane needs to be pretreated using argon plasma to ensure the membrane surface is free from dust particles [134]. In addition, adsorption or polymerization of contaminants can also be avoided by this pre-treatment process.

Basically, CF_4_ plasma treatment can be considered a versatile technique to enhance membrane hydrophobicity without decreasing permeate water flux in the MD process. Woo et al. developed an omniphobic membrane surface by electrospinning followed by CF_4_ plasma treatment [135]. In this study, nanofibrous membranes were treated with CF_4_ plasma and the reduction in surface energy by the growth of new CF_2_–CF_2_ and CF_3_ interactions were observed. After CF_4_ plasma treatment for 15 min under controlled conditions, the average CA improved from 133° to 160°, demonstrating a good enhancement in hydrophobicity. Recently, Lee et al. modified PVDF membranes via plasma treatment followed by hydroxylation of the PVDF membrane surface through the Fenton reaction and growth of microparticles onto the membrane surface. In this case, plasma treatment was used to maximize the surface pore size and further enhance the hydrophobicity of the membrane surface. During this process, a dried PVDF membrane was placed in the plasma apparatus using O_2_ and Ar gases. The PVDF membrane surface was maintained at 150 W for 60 s. The overall outcomes suggest that the modified PVDF membrane with enlarged pore size distribution exhibited stable flux and good antiwetting properties [136].

In another interesting study, the researchers attempted to change the hydrophilic property to hydrophobic property via plasma treatment. Wei et al. used CF_4_ plasma modification technique to transform hydrophilic membranes into a hydrophobic membrane for MD application. The plasma treatment conditions were optimized with respect to the treatment period and plasma glow discharge power utilizing the PES polymeric membrane. During this process, CF_4_ plasma treatment exhibited partial etching along with moderate fluorination effect, which enhanced the hydrophobicity of the membrane surface. The results suggested that high vapor flux has been achieved because of the high porosity of the base membrane [137]. Another study demonstrated the use of plasma treatment for improving the superhydrophobic character of the membrane surface. Tian et al. have fabricated a flat sheet PSF membrane, blended with polyvinylpyrrolidone (PVP) polymers by dual-bath coagulation phase inversion. Superhydrophobic features were achieved after CF4 plasma treatment. This study demonstrated the fabrication of PSF membranes with a high pore interconnective structure and low tortuosity. In addition, the modified membranes showed 2.5 times higher water flux as compared to PVDF commercial membranes when applied in the MD process [138].

The plasma treatment process mainly depends on the following few factors: (a) Monomer type, (b) intensity of lamp, (c) flow rate of gas, (d) operation duration, and € pressure [135,139]. Interestingly, plasma treatment can be utilized for wetting resistance. Hydrophilic PES membranes can be transformed into hydrophobic PES membranes with a significant increment in CA utilizing tetrafluoromethane (CF_4_) plasma modification, which forms a fluorinated layer onto the modified membrane surface. Furthermore, it was observed that tetrafluoromethane plasma treatment allowed a significant improvement in the water CA compared with virgin PE membranes. Table 8 demonstrates the basic advantages of plasma treatment for the fabrication of functionalized hydrophobic membranes for improved MD application. However, for the first time, plasma treatment needs expensive equipment.

##### Chemical Vapor Deposition for Membrane Modification

**Figure 13:**
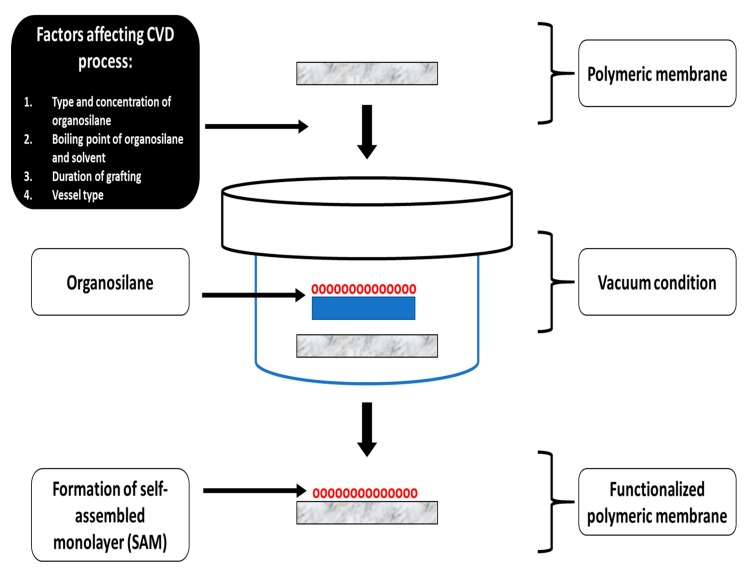
Schematic diagram representing the chemical vapor deposition technique for fabrication of antiwetting and superhydrophobic membrane surfaces.

In this section, chemical vapor deposition (CVD) is thoroughly reviewed. Typically, CVD is a commonly utilized material-processing technology that involves coating of a solid thin film on the desired surface. CVD is a continuous process of chemical reactions involving gaseous reactants and is performed on or near the vicinity of a heated substrate surface. CVD offers structural control at the nanometer scale [144]. CVD can be used for membrane to produce hydrophobic membranes by dip-coating in an organosilane-based solution, as shown in Figure 13. There are certain crucial parameters that must be considered, while executing the CVD process: (1) Organosilane type, (2) organosilane concentration, (3) boiling point of solvent and organosilane, (4) duration of grafting, and (5) vessel type or volume of vessel to be used [145,146]. Although there are numerous advantages of the CVD technique for fabrication of hydrophobic functionalized membranes, it also possesses certain limitations (Table 9).

Warsinger et al. fabricated a superhydrophobic polymeric membrane by initiated chemical vapor deposition (iCVD) of perfluorodecyl acrylate onto PVDF membrane and used the membranes to investigate the effects of surface energy on wetting and fouling propensity. In this study, air layers were maintained onto the membrane surface and significantly minimized fouling. The introduction of air layers was sufficient enough to displace the fouling gels; hence, it decreased the area of contact of the feed stream with the membrane surface, and finally, it improved the superhydrophobic feature in the Cassie–Baxter form. Thus, the combined effect of air recharging and superhydrophobicity minimized fouling onto the membrane surface [147].

Earlier, Huang et al. fabricated a novel Janus membrane to overcome the issue of wetting and fouling in the MD process to desalinate a hypersaline brine solution stream containing both hydrophobic foulants, as well as amphiphilic wetting agents. In this study, a positively charged electrospun nanofibrous layer of CTAB/PVDF-HFP was fabricated, and then, the nanofibrous substrate was coated with SiNPs via dip coating followed by CVD for further fluorination. The SiNPs were fluorinated via CVD by exposing the substrate coated with nanoparticles to 0.15 mL of fluoroalkylsilane under vacuum condition at 100 °C for 1 day. During the MD process, visual inspection revealed that the unmodified PVDF/HFP membrane was significantly fouled after 10 h operation. In contrast, the Janus membranes showed stable performance in terms of water flux and salt rejection in MD operation [148]. In another interesting study, Servi et al. demonstrated the impact of iCVD layering on membrane performance and LEP. The authors confirmed that the overall water flux of MD membranes could be increased when the iCVD film thickness is decreased. In this study, iCVD was utilized, followed by track-etched treatment to fabricate polycarbonate membranes. Furthermore, this dual-layered membrane successfully maximized the LEP value of the fabricated membrane. In addition, the base layer was sized to reduce iCVD film thickness resulting in increased permeability. The author observed that film thickness must be minimized, while focusing on the development of surface coating [149].

Therefore, for fabricating superhydrophobic membrane surfaces, lower surface energy-based component vapors are applied onto the membrane surface to create a top thin, rough layer and antiwetting surface. In addition, CVD can control the crystallinity of the surface coated polymers which are tuned by optimizing the deposition conditions. These above-mentioned optimization conditions over the orientation of the polymer chain can influence the topography of the membrane surface [144].

#### 4.3.3. Overall Perspective

In this section, a brief comparative study of various feasible methodologies for producing antiwetting surfaces is presented (Table 10a,b). Commonly used techniques to generate rough surfaces onto MD membranes for the creation of antiwetting and superhydrophobic membranes include blending or coating methods, electrospinning techniques, phase inversion techniques, plasma treatment, and CVD. These above-mentioned techniques can be categorized into two—wet chemical processes, as well as dry physical methods. Particularly, these techniques are differentiated in terms of surface roughness, operational time duration, and overall outcome.

**Figure 14:**
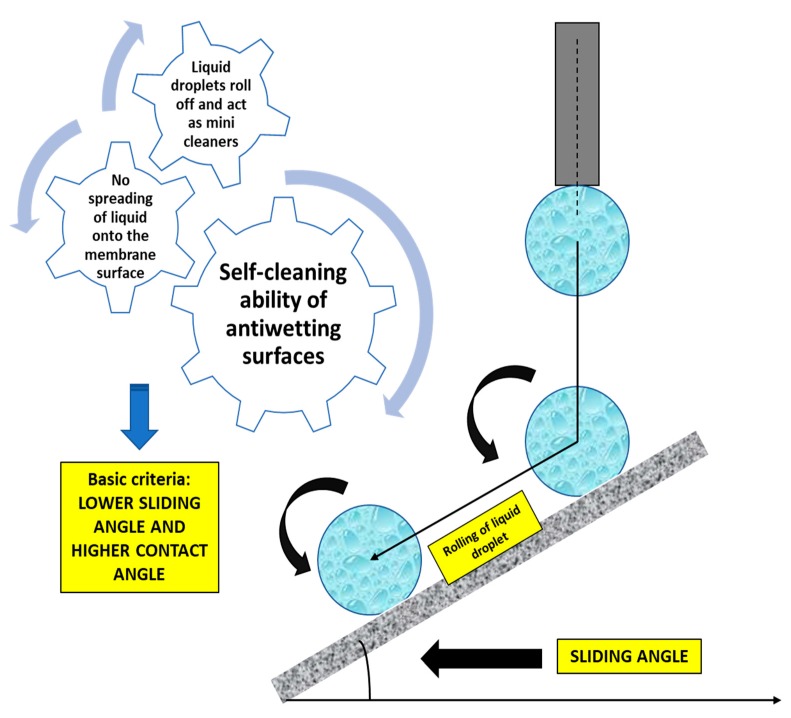
Co-relation of sliding angle and self-cleaning property of an antiwetting membrane surface.

Furthermore, superhydrophobic materials have a huge potential for application in the field of antifouling, antiwetting, and self-cleaning. Recent studies suggest that many novel superhydrophobic membranes have been fabricated with good antifouling properties with incorporated biocidal species or components. The higher the hydrophobicity, the better is the self-cleaning ability because the membrane avoids the spreading of water onto the surface [111,155]. Nature is one of the best sources of inspiration for researchers to fabricate superhydrophobic and self-cleaning functional surfaces for improved MD performance. The self-cleaning features of MD membranes involve a combination of high surface roughness and lower surface energy, which also demonstrates the Lotus effect [156,157]. Thus, the low sliding angle and high-water CA leads to a superhydrophobic membrane surface. Figure 14 demonstrates the self-cleaning mechanism of a superhydrophobic membrane.

In an interesting study, Deng et al. demonstrated a novel hybrid bilayered membrane composed of a superhydrophobic active layer of an amorphous PP and an electrospun nanofibrous PVDF support layer which exhibited a low sliding angle. This bilayered composite membrane was fabricated by a simple vacuum filtration method. The superhydrophobic amorphous PP layer offers an extra barrier to membrane wetting. In addition, the interconnected PVDF nanofibrous layer offers an excellent pathway for vapor flux which results in better DCMD performance. This study attempted to mimic the lotus effect by fabricating a specialized hierarchical structure to demonstrate the self-cleaning ability of the membrane. Notably, the sliding angle of the amorphous PP/PVDF membrane was found to be less than 10°, indicating that the self-cleaning ability of the composite membrane is similar to the lotus effect, which is beneficial for eradicating foulants on the membrane surface. This outcome is due to the combined effect of constructed hierarchical rough surface and low surface energy of amorphous PP. Finally, the composite membrane performed well in terms of stable water flux (50 LMH) and high salt rejection in the DCMD process for 50 h [158].

Now, based on comparative study between phase inversion, electrospinning and mechanical stretching, Table 11 indicates the membrane performance in terms of permeate flux and salt rejection of polymeric membranes fabricated by three various techniques. In short, it can be concluded that, these methodologies seem to be efficient for long term performance as the fabricated membranes could achieve maximum salt rejection.

As far as the selectivity and permeability of MD processes is concerned, the selection of a particular type of MD configuration depends upon the flux, volatility and the permeate composition, whereas, the MD membranes are selected on the basis of their heat transfer and mass transfer characteristics, with selectivity controlled by conventional distillation involving the relative vapor pressures of feed components and operating conditions. Typically, for better selectivity, a hydrophobic porous membrane is tailored so as to have a pore size range of 0.1 to 1µm. Recent research and developments showed excellent selective permeability towards various components. For instance, a sub-micron thin graphene oxide (GO) membrane can retain all gases and liquids through the membrane except water molecules [161]. However, for a given feed stream and membrane type, the wetting tendency can be minimized by choosing proper conditions of temperature and flow rate. Coming to the next point, the prominent barrier in the utilization of polymeric MD membranes in industrial applications is their intrinsic trade-off relationship between selectivity and permeability. Some inorganic membranes, such as zeolite and carbon molecular sieves exhibit maximum permeability and selectivity compared to polymeric membranes, but these inorganic membranes are expensive and pose a difficulty for large-scale manufacturing. Thus, it is recommended to use an alternate cost-effective membrane in a position above the trade-off curves between permeability and selectivity.

## 5. Research Challenges and Future Perspectives

Although MD is a versatile separation process that has been widely utilized at the lab-scale, it has not been implemented at the industrial level. Thus, more research must be focused on scaling up of the MD process, followed by membrane modification for improved MD performance. Antiwetting or superhydrophobic surfaces have considerable potential in terms of water flux and rejection for use in MD applications. With regard to the research challenges, antiwetting or superhydrophobic surfaces need to be designed to be reproducible, scalable, and economic [162,163]. In addition, most available techniques are typically complex and time consuming. Thus, modifying inorganic and phase inversion based polymeric membranes are the best examples. Thus, a more facile approach must be adopted to design antiwetting surfaces for improved MD performance. There are certain concerns involved, while designing antiwetting surfaces:

(1) The durability of superhydrophobic coating must be higher. A facile pathway to design mechanically robust interconnected nanostructures/microstructures with overhanging geometries must be invented.
(2) An easy approach to characterize antiwetting surfaces should be utilized. Even though CA analysis is easy and straight forward, it does not describe the feed-membrane interface in MD application. In addition, CA must be measured in a high temperature environment as the liquid surface tension is closely related to temperature.
(3) The coatings must resist the acidic and basic nature of the feed stream. The properties of coating depend on the chemical nature of the material utilized.
(4) Typically, during long-term operation, surface structures with overhanging features show weaker mechanical resistance because they may influence the surface chemistry, as well as geometry, of the pore structures.
(5) Precise control of surface protrusions, roughness, and geometry is crucial to achieve Cassie stable state for high liquid repellency.
(6) Finally, the surface tension of the feed stream is another major issue, while applying antiwetting surfaces in MD. Furthermore, the operating parameters must be optimized for better performance because a higher water flux (i.e., higher evaporation rate) leads to more severe concentration polarization, and thus, maximizes the chances of wetting.

**Figure 15:**
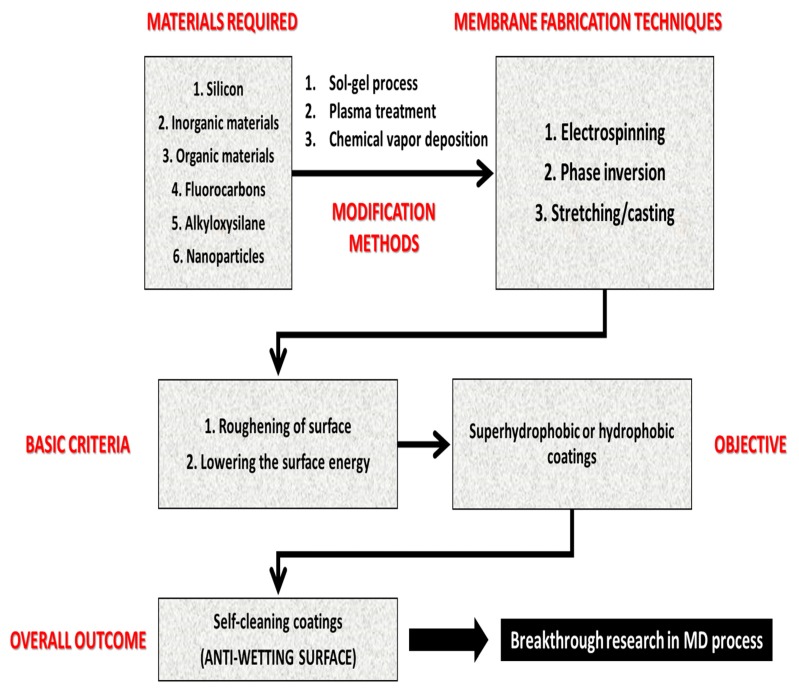
A typical flowchart demonstrating the summary of different materials and processing techniques for achieving antiwetting surfaces for MD process.

Much research have been successful in the practical scenario, but unfortunately, some were unsuccessful because of unavoidable factors, such as (a) peeling off of the layer and (b) change in the chemical composition of the membrane surface after exposure of corrosive feed stream. However, “peeling off” of the superhydrophobic layer from substrates is a major issue that needs to be addressed, while casting antiwetting and self-cleaning membrane surfaces for the MD process. Micro cracks/scratches on the membrane surface must be avoided. The stability of nanoparticles or coatings onto the membrane must be focused on. Therefore, these factors can be considered as topics for future research and development to produce self-cleaning coatings and antiwetting surfaces for more viable commercialization. Figure 15 demonstrates a brief summary of different materials, casting processes, and basic mechanisms involved in the production of self-cleaning coatings for MD application.

Based on current literature available on the MD process and membranes, several future aspects can be explored, such as: Risk of surface and pore wetting must be studied systematically; utilization of low grade heat must be encouraged as MD is economically cheap only when waste heat is available; more research and development is needed for fabrication anti-wetting and anti-fouling MD membranes as limited providers are available; collaborative research work is required between academics and industries as MD needs to be commercialized in industrial scale.

## 6. Conclusions

In this review, an overview of the techniques for fabricating antiwetting or superhydrophobic surfaces for MD operation is presented. The designing of antiwetting membranes not only enhance the self-cleaning properties, but also improves the long-term performance in the MD process. This will have a positive impact on the overall cost of MD process. Therefore, it is essential to cast novel MD membranes with specific features, such as, low resistance to mass transfer, low thermal conductivity, high chemical resistance in order to reduce wetting and fouling tendency. More importantly, these above-mentioned features can influence energy consumption, as well as improve the overall permeate flux and salt rejection. Superhydrophobicity is discussed thoroughly to be utilized as a fundamental basis for creating antiwetting surfaces. The primary aim of this review was the development of antiwetting surfaces with chemical and mechanical stability for better performance in MD application. The science and engineering behind the designing of ultrahydrophobic surfaces were described with high scientific discussions. Preparation techniques for ultrahydrophobic surfaces were reviewed, including surface modification and direct processing methods. Some antiwetting surfaces also demonstrate antifouling characteristics when humid acid and surfactant-based oil/water emulsions are used in the feed stream. As per the present review, chemical and mechanical strength of ultrahydrophobic coatings were found to be a major limitation when MD membranes are exposed to a corrosive environment. The present review could provide a better understanding of creating antiwetting and antifouling surfaces for enhanced MD performance.

## Figures and Tables

**Table 1:** Facile techniques for restoration of wetted MD membranes.

Technique	Working Condition	Overall Outcomes	References
Drying up	Simple air jets are used onto the membrane surface	Minimized surface wetting Salt remains in membrane pores	[12]
Air backwashing	Pressurized air forces water and salt out of pores	Minimized pore and surface wetting	[13]
Chemical treatment	Acid or other chemicals may dissolve the deposits (May involve a drying up technique)	Minimized pore and surface wetting	[14]

**Table 2:** The interrelation between surface tension and wetting of a solid surface and corresponding wetting behavior.

Quantitative Equation	Contact Angle Relationship	Overall Prediction (State)
*γ*_SV_ − *γ*_SL_ > 0	0° ≤ θ ≤ 90°	High wettability
*γ*_SV_ − *γ*_SL_ > *γ*_LV_	θ = 0°	Complete wetting (spreading)
*γ*_SV_ − *γ*_SL_ *<* 0	90° ≤ θ ≤ 180°	Low wetting
*γ*_SL_ − *γ*_SV_ > *γ*_LV_	θ = 180°	No wetting

**Table 3:** Physico-chemical properties of MD membranes with basic requirements for prevention of membrane wetting.

Factors	Conditions	Reference
Layers	At least the top layer must be hydrophobic or superhydrophobic in nature to avoid wetting in the MD process	[48]
Pore size distribution	Selection of a narrow pore size range with a high liquid entry pressure (LEP) value	[32,49,50]
Tortuosity factor	Must be as lower as possible: This is a measure of the deviation in pore structure from normal straight cylindrical pores. Typically, the tortuosity factor is inversely proportional to MD permeability	[4]
Thermal conductivity of the membrane surface	The thermal conductivity of the membrane surface must be as low as possible	[51]
Fouling resistance	The polymeric membrane can be coated with fouling resistant materials to ensure high permeate flux	[52]
Thermal stability	The MD polymeric membrane must show high thermal stability up to 80 °C.	[53]
Chemical resistance	The MD membranes must exhibit good chemical resistance because they may come in contact with acids and bases	[54]

**Table 4:** Effects of membrane wetting on the MD process.

Effects	Reasons	Comments	Reference
Scaling of membrane	Scaling occurs because of the deposition of inorganic ions and components that reduce the hydrophobicity of the MD membrane	Deposition of crystalline inorganic corrosive foulants	[57,60]
Organic and biofouling	Growth of biofoulants reduces the hydrophobicity and surface tension	Interaction between the aqueous medium and hydrophobic surface	[58,61]
Degradation of membrane	Formation of hydrophilic chemical groups, due to long term use of the membrane	Loss of chemical and mechanical stability makes MD process difficult	[62]
Feed stream-based wetting	Surfactant-based feed stream reduces the liquid entry pressure (LEP) value	The liquid entry pressure (LEP) is directly proportional to the surface tension	[62]

**Table 5:** Factors affecting membrane wetting and fouling in the MD process.

Parameters	Factors	Comments	References
Operational conditions	Effect of temperature	Salts, such as CaSO_4_ and CaCO_3_, which have a negative correlation of solubility with respect to temperature also tend to become saturated in the feed stream of desalination. Typically, for commonly utilized feed solutions, increased temperature leads to increased risk of scaling and fouling	[69,70]
	Effect of dissolved gases	The presence of dissolved gases in the feed stream leads to chemical processes, such as the breakdown of bicarbonates which penetrate the membrane pores along with water vapor, exerting an additional diffusive resistance for the water vapor.	[52]
Membrane properties	Thickness of the membrane	Membrane thickness seems to be inversely proportional to the mass and heat transfer rate across the MD membrane. Thus, an optimized membrane thickness must be utilized.	[71]
	Pore size distribution	The pore size of the MD membrane ranges from 0.1 µm to 1 µm In general, pore size affects the mass transfer mechanism. For instance, one side of the membrane, the pore size should be small so that water or liquid cannot penetrate into the pores, while, the pore size must be large on another side, in order to achieve high permeate flux.	[72]
	Porosity	Higher porosity of the membrane offers more water flux, but rapidly wets the membrane	[73]
	Surface energy	A low surface energy offers high hydrophobicity	[74]
Feed solution chemistry	Surface tension	If the feed solution is composed of surfactants higher than the critical value, membrane and pore wetting occurs. Thus, feed streams with a low surface tension must be avoided	[75]
	Concentration of non-volatile solutes	A higher concentration of inorganic salts may result in the formation of salt crystals onto the membrane surface, leading to wetting of membrane portions occupied by salt crystals. Therefore, a higher concentration of inorganic salts may lead to the formation of a cake layer of inorganic foulants that leads to membrane and pore wetting	[76]

**Table 6:** Criteria for waterproof and superhydrophobic electrospun nanofibrous composite membranes for MD application.

Criteria	Description	Reference
Hierarchical structure and lower surface energy	A hierarchical structure blended with lower surface energy materials are required to prevent membrane wetting which occurs because of the presence of a hot feed stream consisting of an NaCl solution.	[89]
Porous skin layer	The top active layer must be porous in nature with proper porosity, pore size range, and interconnected pore channels without blocking the support layer from achieving a high-water flux	[90]
Mechanical and chemical stability	The mechanical and chemical stability between the active layer and support layer must be high, so that it can perform efficiently during the hydraulic impact in MD application	[91]

**Table 7:** State-of-the-art nanoparticle enhanced membranes utilizing various techniques and their potential outcome on MD performance.

Polymeric Solution	Additives or Nanoparticle Type	Overall Outcome	Reference
Polyvinylidene fluoride (PVDF) solution	TiO_2_ nanoparticles	High and stable flux	[114]
PVDF solution	Carbon nanotubes	Flux improvement	[115]
Poly(vinylidene fluoride-co-hexafluoropropylene) (PVDF-HFP) solution	Fluorosilane coated TiO_2_ nanoparticles	Wetting resistance	[114]
PVDF-HFP solution	Graphene	Stable flux	[116]
PVDF solution	Clay particles	Wetting resistance	[117]
PVDF solution	SiO_2_ nanoparticles	Wetting and fouling resistance	[118]
PVDF solution	Multilayer graphene platelets	Improved productivity–efficiency trade-off and enhanced wetting resistance	[119]
PVDF solution	Silver nanoparticles/Multi-walled carbon nanotubes (AgNPs/f-MWCNTs)	High anti-biofouling and separation efficiency	[120]
PVDF-HFP solution	Carbon nanotubes (CNTs)	Superhydrophobicity and efficient desalination performance	[121]
PVDF solution	Metal organic framework: MOF (iron 1,3,5-benzenetricarboxylate)	Stable performance and stable liquid entry pressure	[122]

**Table 8:** Salient features of plasma treatment for the fabrication of functionalized membrane surfaces.

Advantages	Explanation	Reference
Facile approach	1. Simple methodology 2. Formation of uniform, consistent, thin, and clear coating layer	[140]
Strong adhesion	1. Chemically and mechanically stable coating 2. Dense layer formation onto the polymeric surface	[141]
Surface roughening	1. Transformation of hydrophobic to superhydrophobic surface	[142]
Antifouling and antiwetting	1. Membranes with improved fouling resistance can be fabricated 2. Superhydrophobicity can be achieved and results in antiwetting properties	[143]

**Table 9:** Overview of chemical vapor deposition in terms of advantages and disadvantages.

Major Advantages	Limitations
1. Avoids the line of sight	1. Requirement of high temperature
2. High deposition rate	2. High possibility of toxic precursor
3. Production of thick coating layers	3. Mostly inorganic materials can be used
4. Co-deposition of material at same time	

**Table 10:** (**a**): An overview of feasible methodologies to fabricate superhydrophobic polymeric membranes and the overall outcome; (**b**) overview of useful techniques to modify and generate antiwetting and superhydrophobic coatings.

		**Table 10 (a)**		
**Methodologies**	**Casting Parameters**	**Membrane Characteristics**	**Membrane Performance**	**Reference**
Electrospinning technique	Ease: Moderate Replication: Moderate	Pore size distribution: Maximum Surface roughness: Maximum	Overall cost: Moderate Permeate flux: Maximum Wetting tendency: Minimum	[111,150]
Mechanical stretching technique	Ease: Moderate Replication: Moderate	Pore size distribution: Same range Surface roughness: Lower	Overall cost: Moderate Permeate flux: Moderate Wetting tendency: Minimum	[151]
Phase inversion technique	Ease: Maximum Replication: Maximum	Pore size distribution: Same range Surface roughness: Minimum	Overall cost: Minimum Permeate flux: Maximum Wetting tendency: Moderate	[152]
		**Table 10 (b)**		
**Membrane Modification Techniques**	**Modification Process**	**Processing Time**	**Overall Outcome**	**References**
Blending or coating technique	Coated with nanoparticles or nanosols	1. Bit slow	Chemically stable	[153]
Plasma treatment	Growth of nanoparticles by etching	1. Moderate 2. Requires particular equipment	Self-cleaning ability	[138,154]
Chemical vapor deposition	Formation of nanostructures by polymerization	1. Slow 2. Require heating treatment	Good separation efficiency	[79]

**Table 11:** Membrane performance in terms of permeate flux and salt rejection for various membrane fabrication methodologies.

Technique Utilized	Polymer Type	Additive or Agent/Processing	Permeate Flux	Salt Rejection	Reference
**Phase inversion Technique**	Polyvinylidene fluoride (PVDF)	Mechanical scratching	90 LMH	99.99%	[98]
**Phase inversion Technique**	Polyvinylidene fluoride (PVDF)	SiO_2_ nanoparticles	3 kg/m^2^ h	99.98%	[159]
**Electrospinning Technique**	Polyvinylidene fluoride- Polytetrafluoroethylene (PVDF-PTFE)	PTFE micro powders	18.5 kg/m^2^ h	99.9%	[86]
**Electrospinning Technique**	Poly(vinylidene fluoride-co-hexafluoropropylene) (PVDF-HFP)	Graphene	22.9 LMH	100%	[116]
**Mechanical Stretching Method**	Polytetrafluoroethylene (PTFE)	Stretching	5 LMH	99.99%	[160]
**Mechanical Stretching Method**	Polyvinylidene fluoride (PVDF)	Stretching	41.5 kg/m^2^ h	99.99%	[103]

Notes: Conditions, such as module type, concentration of NaCl, temperature difference, and time duration may differ.

## References

[1] Lawson K.W., Lloyd D.R. (1997). Membrane distillation. J. Membr. Sci..

[2] Alkhudhiri A., Darwish N., Hilal N. (2012). Membrane distillation: A comprehensive review. Desalination.

[3] Kaplan R., Mamrosh D., Salih H.H., Dastgheib S.A. (2017). Assessment of desalination technologies for treatment of a highly saline brine from a potential CO_2_ storage site. Desalination.

[4] El-Bourawi M., Ding Z., Ma R., Khayet M. (2006). A framework for better understanding membrane distillation separation process. J. Membr. Sci..

[5] Pangarkar B.L., Sane M.G., Guddad M. (2011). Reverse osmosis and membrane distillation for desalination of groundwater: A review. ISRN Mater. Sci..

[6] Kiss A.A., Kattan Readi O.M. (2018). An industrial perspective on membrane distillation processes. J. Chem. Technol. Biotechnol..

[7] Nawi N.I.M., Bilad M.R., Zolkhiflee N., Nordin N.A.H., Lau W.J., Narkkun T., Faungnawakij K., Arahman N., Mahlia T.M.I. (2019). Development of A Novel Corrugated Polyvinylidene difluoride Membrane via Improved Imprinting Technique for Membrane Distillation. Polymers.

[8] Ray S.S., Chen S.S., Sangeetha D., Chang H.M., Thanh C.N.D., Le Q.H., Ku H.M. (2018). Developments in forward osmosis and membrane distillation for desalination of waters. Environ. Chem. Lett..

[9] Lu K.J., Chen Y., Chung T.-S. (2019). Design of omniphobic interfaces for membrane distillation—A review. Water Res..

[10] Guillen-Burrieza E., Mavukkandy M., Bilad M., Arafat H. (2016). Understanding wetting phenomena in membrane distillation and how operational parameters can affect it. J. Membr. Sci..

[11] Rahimpour M.R., Esmaeilbeig M.A. (2019). Membrane Wetting in Membrane Distillation. Current Trends and Future Developments on (Bio-) Membranes.

[12] Guillen-Burrieza E., Thomas R., Mansoor B., Johnson D., Hilal N., Arafat H. (2013). Effect of dry-out on the fouling of PVDF and PTFE membranes under conditions simulating intermittent seawater membrane distillation (SWMD). J. Membr. Sci..

[13] Warsinger D.M., Servi A., Connors G.B., Mavukkandy M.O., Arafat H.A., Gleason K.K. (2017). Reversing membrane wetting in membrane distillation: Comparing dryout to backwashing with pressurized air. Environ. Sci. Water Res. Technol..

[14] Tijing L.D., Woo Y.C., Choi J.-S., Lee S., Kim S.-H., Shon H.K. (2015). Fouling and its control in membrane distillation—A review. J. Membr. Sci..

[15] Wang S., Li Y., Fei X., Sun M., Zhang C., Li Y., Yang Q., Hong X. (2011). Preparation of a durable superhydrophobic membrane by electrospinning poly (vinylidene fluoride) (PVDF) mixed with epoxy–siloxane modified SiO_2_ nanoparticles: A possible route to superhydrophobic surfaces with low water sliding angle and high water contact angle. J. Colloid Interface Sci..

[16] Cao L., Jones A.K., Sikka V.K., Wu J., Gao D. (2009). Anti-icing superhydrophobic coatings. Langmuir.

[17] Liao Y., Wang R., Fane A.G. (2013). Engineering superhydrophobic surface on poly (vinylidene fluoride) nanofiber membranes for direct contact membrane distillation. J. Membr. Sci..

[18] Mosadegh-Sedghi S., Rodrigue D., Brisson J., Iliuta M.C. (2014). Wetting phenomenon in membrane contactors–causes and prevention. J. Membr. Sci..

[19] Wang B., Zhang Y., Shi L., Li J., Guo Z. (2012). Advances in the theory of superhydrophobic surfaces. J. Mater. Chem..

[20] Gao N., Yan Y. (2009). Modeling superhydrophobic contact angles and wetting transition. J. Bionic Eng..

[21] Whyman G., Bormashenko E., Stein T. (2008). The rigorous derivation of Young, Cassie–Baxter and Wenzel equations and the analysis of the contact angle hysteresis phenomenon. Chem. Phys. Lett..

[22] Good R.J. (1992). Contact angle, wetting, and adhesion: A critical review. J. Adhes. Sci. Technol..

[23] Eick J., Good R., Neumann A. (1975). Thermodynamics of contact angles. II. Rough solid surfaces. J. Colloid Interface Sci..

[24] Li D., Neumann A. (1992). Contact angles on hydrophobic solid surfaces and their interpretation. J. Colloid Interface Sci..

[25] Li W., Amirfazli A. (2005). A thermodynamic approach for determining the contact angle hysteresis for superhydrophobic surfaces. J. Colloid Interface Sci..

[26] Zuo Y.Y., Do C., Neumann A.W. (2007). Automatic measurement of surface tension from noisy images using a component labeling method. Colloids Surf. A Physicochem. Eng. Asp..

[27] Dorrer C., Rühe J. (2009). Some thoughts on superhydrophobic wetting. Soft Matter.

[28] Butt H.-J., Roisman I.V., Brinkmann M., Papadopoulos P., Vollmer D., Semprebon C. (2014). Characterization of super liquid-repellent surfaces. Curr. Opin. Colloid Interface Sci..

[29] Zhu J., Dai X. (2019). A new model for contact angle hysteresis of superhydrophobic surface. AIP Adv..

[30] Chen W., Fadeev A.Y., Hsieh M.C., Öner D., Youngblood J., McCarthy T.J. (1999). Ultrahydrophobic and ultralyophobic surfaces: Some comments and examples. Langmuir.

[31] Murase H., Nanishi K., Kogure H., Fujibayashi T., Tamura K., Haruta N. (1994). Interactions between heterogeneous surfaces of polymers and water. J. Appl. Polym. Sci..

[32] Franken A., Nolten J., Mulder M., Bargeman D., Smolders C. (1987). Wetting criteria for the applicability of membrane distillation. J. Membr. Sci..

[33] Jung Y.C., Bhushan B. (2009). Wetting behavior of water and oil droplets in three-phase interfaces for hydrophobicity/philicity and oleophobicity/philicity. Langmuir.

[34] Nosonovsky M. (2007). On the range of applicability of the Wenzel and Cassie equations. Langmuir.

[35] Gao L., McCarthy T.J. (2007). How Wenzel and Cassie were wrong. Langmuir.

[36] Murakami D., Jinnai H., Takahara A. (2014). Wetting transition from the Cassie–Baxter state to the Wenzel state on textured polymer surfaces. Langmuir.

[37] Jung Y.C., Bhushan B. (2007). Wetting transition of water droplets on superhydrophobic patterned surfaces. Scr. Mater..

[38] Vrancken R.J., Kusumaatmaja H., Hermans K., Prenen A.M., Pierre-Louis O., Bastiaansen C.W., Broer D.J. (2009). Fully reversible transition from Wenzel to Cassie−Baxter states on corrugated superhydrophobic surfaces. Langmuir.

[39] Pan Z., Cheng F., Zhao B. (2017). Bio-inspired polymeric structures with special wettability and their applications: An overview. Polymers.

[40] Iqbal M., Dinh D.K., Abbas Q., Imran M., Sattar H., Ul Ahmad A. (2019). Controlled Surface Wettability by Plasma Polymer Surface Modification. Surfaces.

[41] Mohamed A.M., Abdullah A.M., Younan N.A. (2015). Corrosion behavior of superhydrophobic surfaces: A review. Arab. J. Chem..

[42] Milne A., Amirfazli A. (2012). The Cassie equation: How it is meant to be used. Adv. Colloid Interface Sci..

[43] Lu X., Peng Y., Qiu H., Liu X., Ge L. (2017). Anti-fouling membranes by manipulating surface wettability and their anti-fouling mechanism. Desalination.

[44] Ray S.S., Gandhi M., Chen S.-S., Chang H.-M., Dan C.T.N., Le H.Q. (2018). Anti-wetting behaviour of a superhydrophobic octadecyltrimethoxysilane blended PVDF/recycled carbon black composite membrane for enhanced desalination. Environ. Sci. Water Res. Technol..

[45] Fürstner R., Barthlott W., Neinhuis C., Walzel P. (2005). Wetting and self-cleaning properties of artificial superhydrophobic surfaces. Langmuir.

[46] Koishi T., Yasuoka K., Fujikawa S., Zeng X.C. (2011). Measurement of contact-angle hysteresis for droplets on nanopillared surface and in the Cassie and Wenzel states: A molecular dynamics simulation study. ACS Nano.

[47] Marmur A. (2003). Wetting on hydrophobic rough surfaces: To be heterogeneous or not to be?. Langmuir.

[48] Khayet M., Matsuura T. (2011). Membrane Distillation: Principles and Applications.

[49] Drioli E., Ali A., Macedonio F. (2015). Membrane distillation: Recent developments and perspectives. Desalination.

[50] Dommati H., Ray S.S., Wang J.C., Chen S.S.A. (2019). A comprehensive review of recent developments in 3D printing technique for ceramic membrane fabrication for water purification. RSC Adv..

[51] Subramanian N., Qamar A., Alsaadi A., Gallo A., Ridwan M.G., Lee J.G., Pillai S., Arunachalam S., Anjum D., Sharipov F. (2019). Evaluating the potential of superhydrophobic nanoporous alumina membranes for direct contact membrane distillation. J. Colloid Interface Sci..

[52] Warsinger D.M., Swaminathan J., Guillen-Burrieza E., Arafat H.A., Lienhard V J.H. (2015). Scaling and fouling in membrane distillation for desalination applications: A review. Desalination.

[53] Eykens L., De Sitter K., Dotremont C., Pinoy L., Van der Bruggen B. (2016). How to optimize the membrane properties for membrane distillation: A review. Ind. Eng. Chem. Res..

[54] Luo A., Lior N. (2016). Critical review of membrane distillation performance criteria. Desalin. Water Treat..

[55] Goh S., Zhang J., Liu Y., Fane A.G. (2013). Fouling and wetting in membrane distillation (MD) and MD-bioreactor (MDBR) for wastewater reclamation. Desalination.

[56] Nguyen Q.-M., Lee S. (2015). Fouling analysis and control in a DCMD process for SWRO brine. Desalination.

[57] Naidu G., Jeong S., Vigneswaran S., Hwang T.-M., Choi Y.-J., Kim S.-H. (2016). A review on fouling of membrane distillation. Desalin. Water Treat..

[58] Naidu G., Jeong S., Kim S.-J., Kim I.S., Vigneswaran S. (2014). Organic fouling behavior in direct contact membrane distillation. Desalination.

[59] Rezaei M., Warsinger D.M., Duke M.C., Matsuura T., Samhaber W.M. (2018). Wetting phenomena in membrane distillation: Mechanisms, reversal, and prevention. Water Res..

[60] Gryta M. (2008). Fouling in direct contact membrane distillation process. J. Membr. Sci..

[61] Tow E.W., Warsinger D.M., Trueworthy A.M., Swaminathan J., Thiel G.P., Zubair S.M., Myerson A.S. (2018). Comparison of fouling propensity between reverse osmosis, forward osmosis, and membrane distillation. J. Membr. Sci..

[62] Gryta M., Grzechulska-Damszel J., Markowska A., Karakulski K. (2009). The influence of polypropylene degradation on the membrane wettability during membrane distillation. J. Membr. Sci..

[63] Tijing L.D., Woo Y.C., Shim W.-G., He T., Choi J.-S., Kim S.-H., Shon H.K. (2016). Superhydrophobic nanofiber membrane containing carbon nanotubes for high-performance direct contact membrane distillation. J. Membr. Sci..

[64] Ray S.S., Chen S.-S., Chang H.-M., Thanh C.N.D., Le H.Q., Nguyen N.C. (2018). Enhanced desalination using a three-layer OTMS based superhydrophobic membrane for a membrane distillation process. RSC Adv..

[65] Guillén-Burrieza E., Blanco J., Zaragoza G., Alarcón D.-C., Palenzuela P., Ibarra M., Gernjak W. (2011). Experimental analysis of an air gap membrane distillation solar desalination pilot system. J. Membr. Sci..

[66] Guillen-Burrieza E., Servi A., Lalia B.S., Arafat H.A. (2015). Membrane structure and surface morphology impact on the wetting of MD membranes. J. Membr. Sci..

[67] Gilron J., Ladizansky Y., Korin E. (2013). Silica fouling in direct contact membrane distillation. Ind. Eng. Chem. Res..

[68] Curcio E., Drioli E. (2005). Membrane distillation and related operations—A review. Sep. Purif. Rev..

[69] Lawson K.W., Lloyd D.R. (1996). Membrane distillation. II. Direct contact MD. J. Membr. Sci..

[70] Schofield R., Fane A., Fell C., Macoun R. (1990). Factors affecting flux in membrane distillation. Desalination.

[71] Ray S.S., Deb C.K., Chang H.M., Chen S.S., Ganesapillai M. (2019). Crosslinked PVDF-HFP-based hydrophobic membranes incorporated with CNF for enhanced stability and permeability in membrane distillation. J. Appl. Polym. Sci..

[72] Gryta M. (2005). Long-term performance of membrane distillation process. J. Membr. Sci..

[73] Gryta M. (2007). Influence of polypropylene membrane surface porosity on the performance of membrane distillation process. J. Membr. Sci..

[74] Efome J.E., Rana D., Matsuura T., Lan C.Q. (2016). Enhanced performance of PVDF nanocomposite membrane by nanofiber coating: A membrane for sustainable desalination through MD. Water Res..

[75] Eykens L., De Sitter K., Dotremont C., De Schepper W., Pinoy L., Van Der Bruggen B. (2017). Wetting resistance of commercial membrane distillation membranes in waste streams containing surfactants and oil. Appl. Sci..

[76] Shirazi M.M.A., Kargari A., Tabatabaei M. (2014). Evaluation of commercial PTFE membranes in desalination by direct contact membrane distillation. Chem. Eng. Process. Process. Intensif..

[77] Tijing L.D., Choi J.-S., Lee S., Kim S.-H., Shon H.K. (2014). Recent progress of membrane distillation using electrospun nanofibrous membrane. J. Membr. Sci..

[78] De Zarate J.O., Pen L., Mengual J.I. (1995). Characterization of membrane distillation membranes prepared by phase inversion. Desalination.

[79] Ma M., Mao Y., Gupta M., Gleason K.K., Rutledge G.C. (2005). Superhydrophobic fabrics produced by electrospinning and chemical vapor deposition. Macromolecules.

[80] Xu W., Ning T., Yang X., Lu S. (2011). Fabrication of superhydrophobic surfaces on zinc substrates. Appl. Surf. Sci..

[81] Blanco M., Monteserín C., Angulo A., Pérez-Márquez A., Maudes J., Murillo N., Aranzabe E., Ruiz-Rubio L., Vilas J.L. (2019). TiO_2_-Doped Electrospun Nanofibrous Membrane for Photocatalytic Water Treatment. Polymers.

[82] Liu Z., Qin D., Zhao J., Feng Q., Li Z., Bai H., Sun D.D. (2019). Efficient Oil/Water Separation Membrane Derived from Super-Flexible and Superhydrophilic Core–Shell Organic/Inorganic Nanofibrous Architectures. Polymers.

[83] Ray S.S., Chen S.-S., Li C.-W., Nguyen N.C., Nguyen H.T. (2016). A comprehensive review: Electrospinning technique for fabrication and surface modification of membranes for water treatment application. RSC Adv..

[84] Ray S.S., Chen S.-S., Hsu H.-T., Cao D.-T., Nguyen H.-T., Nguyen N.C. (2017). Uniform hydrophobic electrospun nanofibrous layer composed of polysulfone and sodium dodecyl sulfate for improved desalination performance. Sep. Purif. Technol..

[85] Khayet M., García-Payo C., Matsuura T. (2019). Superhydrophobic nanofibers electrospun by surface segregating fluorinated amphiphilic additive for membrane distillation. J. Membr. Sci..

[86] Dong Z.-Q., Ma X.-H., Xu Z.-L., You W.-T., Li F.-B. (2014). Superhydrophobic PVDF–PTFE electrospun nanofibrous membranes for desalination by vacuum membrane distillation. Desalination.

[87] Su C., Li Y., Dai Y., Gao F., Tang K., Cao H. (2016). Fabrication of three-dimensional superhydrophobic membranes with high porosity via simultaneous electrospraying and electrospinning. Mater. Lett..

[88] An X., Liu Z., Hu Y. (2018). Amphiphobic surface modification of electrospun nanofibrous membranes for anti-wetting performance in membrane distillation. Desalination.

[89] Li X., Wang C., Yang Y., Wang X., Zhu M., Hsiao B.S. (2014). Dual-biomimetic superhydrophobic electrospun polystyrene nanofibrous membranes for membrane distillation. ACS Appl. Mater. Interfaces.

[90] Liao Y., Loh C.-H., Wang R., Fane A.G. (2014). Electrospun superhydrophobic membranes with unique structures for membrane distillation. ACS Appl. Mater. Interfaces.

[91] Nuraje N., Khan W.S., Lei Y., Ceylan M., Asmatulu R. (2013). Superhydrophobic electrospun nanofibers. J. Mater. Chem. A.

[92] Zhang W., Shi Z., Zhang F., Liu X., Jin J., Jiang L. (2013). Superhydrophobic and superoleophilic PVDF membranes for effective separation of water-in-oil emulsions with high flux. Adv. Mater..

[93] Yao M., Woo Y.C., Tijing L.D., Shim W.G., Choi J.S., Kim S.H., Shon H.K. (2016). Effect of heat-press conditions on electrospun membranes for desalination by direct contact membrane distillation. Desalination.

[94] Lalia B.S., Kochkodan V., Hashaikeh R., Hilal N. (2013). A review on membrane fabrication: Structure, properties and performance relationship. Desalination.

[95] Liu S., Li K., Hughes R. (2003). Preparation of porous aluminium oxide (Al_2_O_3_) hollow fibre membranes by a combined phase-inversion and sintering method. Ceram. Int..

[96] Eykens L., De Sitter K., Dotremont C., Pinoy L., Van der Bruggen B. (2017). Membrane synthesis for membrane distillation: A review. Sep. Purif. Technol..

[97] Peng M., Li H., Wu L., Zheng Q., Chen Y., Gu W. (2005). Porous poly (vinylidene fluoride) membrane with highly hydrophobic surface. J. Appl. Polym. Sci..

[98] Munirasu S., Banat F., Durrani A.A., Haija M.A. (2017). Intrinsically superhydrophobic PVDF membrane by phase inversion for membrane distillation. Desalination.

[99] Wu C., Tang W., Zhang J., Liu S., Wang Z., Wang X., Lu X. (2017). Preparation of super-hydrophobic PVDF membrane for MD purpose via hydroxyl induced crystallization-phase inversion. J. Membr. Sci..

[100] Xiao Z., Zheng R., Liu Y., He H., Yuan X., Ji Y., Li D., Yin H., Zhang Y., Li X.-M. (2019). Slippery for scaling resistance in membrane distillation: A novel porous micropillared superhydrophobic surface. Water Res..

[101] Ahmed F.E., Lalia B.S., Hashaikeh R. (2015). A review on electrospinning for membrane fabrication: Challenges and applications. Desalination.

[102] Yuan Z., Chen H., Zhang J., Zhao D., Liu Y., Zhou X., Li S., Shi P., Tang J., Chen X. (2008). Preparation and characterization of self-cleaning stable superhydrophobic linear low-density polyethylene. Sci. Technol. Adv. Mater..

[103] Wang K.Y., Chung T.S., Gryta M. (2008). Hydrophobic PVDF hollow fiber membranes with narrow pore size distribution and ultra-thin skin for the fresh water production through membrane distillation. Chem. Eng. Sci..

[104] Kurumada K.-I., Kitamura T., Fukumoto N., Oshima M., Tanigaki M., Kanazawa S.-I. (1998). Structure generation in PTFE porous membranes induced by the uniaxial and biaxial stretching operations. J. Membr. Sci..

[105] Huang L.-T., Hsu P.-S., Kuo C.-Y., Chen S.-C., Lai J.-Y. (2008). Pore size control of PTFE membranes by stretch operation with asymmetric heating system. Desalination.

[106] Huang Q.-L., Xiao C.F., Feng X.S., Hu X.Y. (2013). Design of super-hydrophobic microporous polytetrafluoroethylene membranes. New J. Chem..

[107] Fang H., Gao J.F., Wang H.T., Chen C.S. (2012). Hydrophobic porous alumina hollow fiber for water desalination via membrane distillation process. J. Membr. Sci..

[108] Zhang J., Song Z., Li B., Wang Q., Wang S. (2013). Fabrication and characterization of superhydrophobic poly (vinylidene fluoride) membrane for direct contact membrane distillation. Desalination.

[109] Xu Z., Liu Z., Song P., Xiao C. (2017). Fabrication of super-hydrophobic polypropylene hollow fiber membrane and its application in membrane distillation. Desalination.

[110] Razmjou A., Arifin E., Dong G., Mansouri J., Chen V. (2012). Superhydrophobic modification of TiO2 nanocomposite PVDF membranes for applications in membrane distillation. J. Membr. Sci..

[111] Ma M., Hill R.M., Lowery J.L., Fridrikh S.V., Rutledge G.C. (2005). Electrospun poly (styrene-block-dimethylsiloxane) block copolymer fibers exhibiting superhydrophobicity. Langmuir.

[112] Wang Y., He G., Shao Y., Zhang D., Ruan X., Xiao W., Li X., Wu X., Jiang X. (2019). Enhanced performance of superhydrophobic polypropylene membrane with modified antifouling surface for high salinity water treatment. Sep. Purif. Technol..

[113] Liao Y., Loh C.-H., Tian M., Wang R., Fane A.G. (2018). Progress in electrospun polymeric nanofibrous membranes for water treatment: Fabrication, modification and applications. Prog. Polym. Sci..

[114] Lee E.-J., An A.K., He T., Woo Y.C., Shon H.K. (2016). Electrospun nanofiber membranes incorporating fluorosilane-coated TiO2 nanocomposite for direct contact membrane distillation. J. Membr. Sci..

[115] Gethard K., Sae-Khow O., Mitra S. (2010). Water desalination using carbon-nanotube-enhanced membrane distillation. ACS Appl. Mater. Interfaces.

[116] Woo Y.C., Tijing L.D., Shim W.-G., Choi J.-S., Kim S.-H., He T., Drioli E., Shon H.K. (2016). Water desalination using graphene-enhanced electrospun nanofiber membrane via air gap membrane distillation. J. Membr. Sci..

[117] Prince J., Singh G., Rana D., Matsuura T., Anbharasi V., Shanmugasundaram T. (2012). Preparation and characterization of highly hydrophobic poly (vinylidene fluoride)–Clay nanocomposite nanofiber membranes (PVDF–clay NNMs) for desalination using direct contact membrane distillation. J. Membr. Sci..

[118] Kim Y.J., Ahn C.H., Choi M.O. (2010). Effect of thermal treatment on the characteristics of electrospun PVDF− silica composite nanofibrous membrane. Eur. Polym. J..

[119] Gontarek E., Macedonio F., Militano F., Giorno L., Lieder M., Politano A., Drioli E., Gugliuzza A. (2019). Adsorption-assisted transport of water vapour in super-hydrophobic membranes filled with multilayer graphene platelets. Nanoscale.

[120] Nthunya L.N., Gutierrez L., Khumalo N., Derese S., Mamba B.B., Verliefde A.R., Mhlanga S.D. (2019). Superhydrophobic PVDF nanofibre membranes coated with an organic fouling resistant hydrophilic active layer for direct-contact membrane distillation. Colloids Surf. A Physicochem. Eng. Asp..

[121] An A.K., Lee E.J., Guo J., Jeong S., Lee J.G., Ghaffour N. (2017). Enhanced vapor transport in membrane distillation via functionalized carbon nanotubes anchored into electrospun nanofibres. Sci. Rep..

[122] Yang F., Efome J.E., Rana D., Matsuura T., Lan C. (2018). Metal–organic frameworks supported on nanofiber for desalination by direct contact membrane distillation. ACS Appl. Mater. Interfaces.

[123] Laganà F., Barbieri G., Drioli E. (2000). Direct contact membrane distillation: Modelling and concentration experiments. J. Membr. Sci..

[124] Gnus M., Dudek G., Turczyn R. (2018). The influence of filler type on the separation properties of mixed-matrix membranes. Chem. Pap..

[125] Silva T.L., Morales-Torres S., Figueiredo J.L., Silva A.M. (2015). Multi-walled carbon nanotube/PVDF blended membranes with sponge-and finger-like pores for direct contact membrane distillation. Desalination.

[126] Qtaishat M., Khayet M., Matsuura T. (2009). Guidelines for preparation of higher flux hydrophobic/hydrophilic composite membranes for membrane distillation. J. Membr. Sci..

[127] Wang K.Y., Foo S.W., Chung T.S. (2009). Mixed matrix PVDF hollow fiber membranes with nanoscale pores for desalination through direct contact membrane distillation. Ind. Eng. Chem. Res..

[128] Castro-Muñoz R., Iglesia O.D.L., Fíla V., Téllez C., Coronas J. (2018). Pervaporation-assisted esterification reactions by means of mixed matrix membranes. Ind. Eng. Chem. Res..

[129] Weigelt F., Georgopanos P., Shishatskiy S., Filiz V., Brinkmann T., Abetz V. (2018). Development and characterization of defect-free matrimid^®^ mixed-matrix membranes containing activated carbon particles for gas separation. Polymers.

[130] Zuo J., Chung T.S. (2016). Metal–organic framework-functionalized alumina membranes for vacuum membrane distillation. Water.

[131] Eykens L., De Sitter K., Dotremont C., Pinoy L., Van der Bruggen B. (2018). Coating techniques for membrane distillation: An experimental assessment. Sep. Purif. Technol..

[132] Li Y., Wang L.a., Hu X., Jin P., Song X. (2018). Surface modification to produce superhydrophobic hollow fiber membrane contactor to avoid membrane wetting for biogas purification under pressurized conditions. Sep. Purif. Technol..

[133] Kim H.I., Kim S.S. (2006). Plasma treatment of polypropylene and polysulfone supports for thin film composite reverse osmosis membrane. J. Membr. Sci..

[134] Kawakami M., Yamashita Y., Iwamoto M., Kagawa S. (1984). Modification of gas permeabilities of polymer membranes by plasma coating. J. Membr. Sci..

[135] Woo Y.C., Chen Y., Tijing L.D., Phuntsho S., He T., Choi J.-S., Kim S.-H., Shon H.K. (2017). CF4 plasma-modified omniphobic electrospun nanofiber membrane for produced water brine treatment by membrane distillation. J. Membr. Sci..

[136] Lee H.K., Kim W., Kim Y.M., Kwon Y.-N. (2019). Surface modification of polyvinylidene fluoride membrane for enhanced wetting resistance. Appl. Surf. Sci..

[137] Wei X., Zhao B., Li X.-M., Wang Z., He B.-Q., He T., Jiang B. (2012). CF4 plasma surface modification of asymmetric hydrophilic polyethersulfone membranes for direct contact membrane distillation. J. Membr. Sci..

[138] Tian M., Yin Y., Yang C., Zhao B., Song J., Liu J., Li X.-M., He T. (2015). CF4 plasma modified highly interconnective porous polysulfone membranes for direct contact membrane distillation (DCMD). Desalination.

[139] Zuo G., Wang R. (2013). Novel membrane surface modification to enhance anti-oil fouling property for membrane distillation application. J. Membr. Sci..

[140] Choi J., Lee K.H., Yang S. (2011). Fabrication of PDMS through-holes using the MIMIC method and the surface treatment by atmospheric-pressure CH4/He RF plasma. J. Micromech. Microeng..

[141] Kim H.I., Kim S.S. (2001). Fabrication of reverse osmosis membrane via low temperature plasma polymerization. J. Membr. Sci..

[142] Choi J.H., Jegal J., Kim W.N. (2006). Fabrication and characterization of multi-walled carbon nanotubes/polymer blend membranes. J. Membr. Sci..

[143] Kaur S., Ma Z., Gopal R., Singh G., Ramakrishna S., Matsuura T. (2007). Plasma-induced graft copolymerization of poly (methacrylic acid) on electrospun poly (vinylidene fluoride) nanofiber membrane. Langmuir.

[144] Zheng Z., Gu Z., Huo R., Ye Y. (2009). Superhydrophobicity of polyvinylidene fluoride membrane fabricated by chemical vapor deposition from solution. Appl. Surf. Sci..

[145] Guo F., Servi A., Liu A., Gleason K.K., Rutledge G.C. (2015). Desalination by membrane distillation using electrospun polyamide fiber membranes with surface fluorination by chemical vapor deposition. ACS Appl. Mater. Interfaces.

[146] Ruiz-Vargas C.S., Zhuang H.L., Huang P.Y., Van Der Zande A.M., Garg S., McEuen P.L., Muller D.A., Hennig R.G., Park J. (2011). Softened elastic response and unzipping in chemical vapor deposition graphene membranes. Nano Lett..

[147] Warsinger D.M., Servi A., Van Belleghem S., Gonzalez J., Swaminathan J., Kharraz J., Chung H.W., Arafat H.A., Gleason K.K. (2016). Combining air recharging and membrane superhydrophobicity for fouling prevention in membrane distillation. J. Membr. Sci..

[148] Huang Y.-X., Wang Z., Jin J., Lin S. (2017). Novel Janus membrane for membrane distillation with simultaneous fouling and wetting resistance. Environ. Sci. Technol..

[149] Servi A.T., Guillen-Burrieza E., Warsinger D.M., Livernois W., Notarangelo K., Kharraz J., Arafat H.A., Gleason K.K. (2017). The effects of iCVD film thickness and conformality on the permeability and wetting of MD membranes. J. Membr. Sci..

[150] Ray S.S., Chen S.-S., Nguyen N.C., Nguyen H.T. (2019). Electrospinning: A Versatile Fabrication Technique for Nanofibrous Membranes for Use in Desalination. Nanoscale Materials in Water Purification.

[151] Bonyadi S., Chung T.S. (2007). Flux enhancement in membrane distillation by fabrication of dual layer hydrophilic–hydrophobic hollow fiber membranes. J. Membr. Sci..

[152] Haponska M., Trojanowska A., Nogalska A., Jastrzab R., Gumi T., Tylkowski B. (2017). PVDF membrane morphology—Influence of polymer molecular weight and preparation temperature. Polymers.

[153] Rao A.V., Latthe S.S., Nadargi D.Y., Hirashima H., Ganesan V. (2009). Preparation of MTMS based transparent superhydrophobic silica films by sol–gel method. J. Colloid Interface Sci..

[154] Yang C., Li X.-M., Gilron J., Kong D.-F., Yin Y., Oren Y., Linder C., He T. (2014). CF4 plasma-modified superhydrophobic PVDF membranes for direct contact membrane distillation. J. Membr. Sci..

[155] Ganesh V.A., Raut H.K., Nair A.S., Ramakrishna S.A. (2011). A review on self-cleaning coatings. J. Mater. Chem..

[156] An A.K., Guo J., Lee E.-J., Jeong S., Zhao Y., Wang Z., Leiknes T. (2017). PDMS/PVDF hybrid electrospun membrane with superhydrophobic property and drop impact dynamics for dyeing wastewater treatment using membrane distillation. J. Membr. Sci..

[157] Li X., García-Payo M., Khayet M., Wang M., Wang X. (2017). Superhydrophobic polysulfone/polydimethylsiloxane electrospun nanofibrous membranes for water desalination by direct contact membrane distillation. J. Membr. Sci..

[158] Deng L., Li P., Liu K., Wang X., Hsiao B.S. (2019). Robust superhydrophobic dual layer nanofibrous composite membranes with a hierarchically structured amorphous polypropylene skin for membrane distillation. J. Mater. Chem. A.

[159] Efome J.E., Baghbanzadeh M., Rana D., Matsuura T., Lan C.Q. (2015). Effects of superhydrophobic SiO_2_ nanoparticles on the performance of PVDF flat sheet membranes for vacuum membrane distillation. Desalination.

[160] Li K., Zhang Y., Xu L., Zeng F., Hou D., Wang J. (2018). Optimizing stretching conditions in fabrication of PTFE hollow fiber membrane for performance improvement in membrane distillation. J. Membr. Sci..

[161] Nair R., Wu H.A., Jayaram P.N., Grigorieva I.V., Geim A.K. (2012). Unimpeded permeation of water through helium-leak–tight graphene-based membranes. Science.

[162] Jeevahan J., Chandrasekaran M., Joseph G.B., Durairaj R., Mageshwaran G. (2018). Superhydrophobic surfaces: A review on fundamentals, applications, and challenges. J. Coat. Technol. Res..

[163] Deshmukh A., Boo C., Karanikola V., Lin S., Straub A.P., Tong T., Warsinger D.M., Elimelech M. (2018). Membrane distillation at the water-energy nexus: Limits, opportunities, and challenges. Energy Environ. Sci..

